# Evolutionary trajectories of teleost olfactory signaling genes shaped by long-term redundancy after whole-genome duplication

**DOI:** 10.1016/j.isci.2026.116564

**Published:** 2026-06-30

**Authors:** Tatsuki Nagasawa, Hanami Fujisaki, Takahiro Ogo, Masato Nikaido

**Affiliations:** 1 School of Life Science and Technology, Institute of Science Tokyo, Meguro-ku, Tokyo 152-8550, Japan

**Keywords:** evolution, whole genome duplication, teleost, olfaction

## Abstract

Whole-genome duplication (WGD) facilitates molecular and species diversification. Here, we investigated olfactory marker protein (*omp*) genes duplicated by the teleost-specific WGD through phylogenetic, syntenic, expression, and promoter analyses. Our results suggest that duplicated *omp* genes were retained over an extended evolutionary period spanning approximately 300 million years and underwent both non- and sub-functionalization. Comparisons with the host gene *capn5* and cross-species reporter assays further support regulation by short upstream regions and suggest sequence-level divergence after WGD. In addition, analyses of multiple olfactory signal transduction genes revealed coordinated long-term retention of duplicated gene pairs across the signaling cascade, consistent with gene dosage constraints and prolonged functional redundancy after WGD. These findings imply that the teleost olfactory system retained ancestral redundancy over exceptionally long evolutionary timescales, thereby facilitating regulatory and functional diversification within the olfactory signaling pathway.

## Highlights

• Teleost-specific WGD generated duplicated omp genes that persisted for ∼300 million years
• Extended redundancy in ompa/ompb led to both non- and sub-functionalization
• Olfactory transduction genes also show long-term redundancy shaped by dosage constraints
• WGD likely diversified teleost olfactory signaling via dosage constraints

## Graphical abstract

**Figure:**
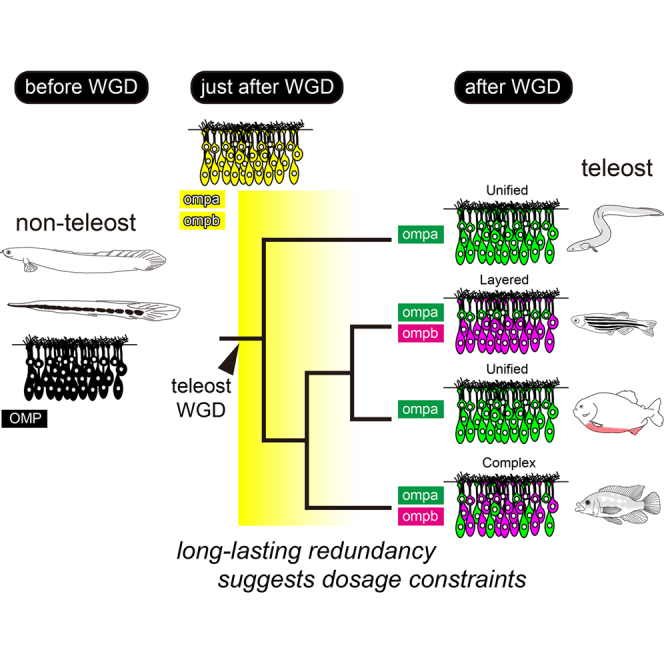

## Teaser

Zoology; evolutionary biology; phylogenetics; phylogeny

## Introduction

Dr. Susumu Ohno proposed a seminal theory for molecular evolution: i.e., “gene duplication is one of the most critical driving forces for molecular evolution.”^1^ Immediately after duplication, gene pairs are in a fully redundant state because they share completely identical sequences, expression patterns, and functions. This redundancy enables one copy to preserve the ancestral function, thereby allowing the other to accumulate mutations under relaxed selective constraints. Consequently, in most duplicated gene pairs, one copy is eventually lost through pseudogenization (non-functionalization), returning to a single-copy state. However, a minority of pairs escape this fate and instead undergo neo-functionalization (acquiring of novel functions) or sub-functionalization (partitioning of ancestral functions). Such divergence has been linked to the acquisition of novel traits^2^^,^^3^ and adaptation to new ecological niches,^4^^,^^5^ and has therefore positioned gene duplication as a key mechanism underlying evolutionary innovation. In particular, whole-genome duplication (WGD) is a transformative evolutionary event, creating duplicates of the entire coding gene repertoire and reshaping genome architecture. Independent WGD events have occurred in major lineages such as plants,^6^^,^^7^ yeast,^8^^,^^9^ and vertebrates,^10^^,^^11^^,^^12^^,^^13^ and are widely regarded as contributing to adaptive radiation.^14^

Teleost fishes—the largest and most diverse vertebrate group—also experienced an additional WGD approximately 300 million years ago (MYA), known as the teleost-specific or third-round (3 R) WGD.^15^^,^^16^^,^^17^^,^^18^ This duplication followed two earlier rounds of genome duplication: the first (1 R) in the ancestor of vertebrates and the second (2 R) in the ancestor of jawed vertebrates.^19^^,^^20^ The teleost-WGD contributed to the evolution of specialized morphologies such as myogenic electric organs^21^ and the bulbus arteriosus,^22^ facilitating the remarkable ecological expansion of teleosts across diverse aquatic environments. Comparative genomic analyses have revealed that WGD-derived gene pairs follow a two-phase evolutionary trajectory.^23^^,^^24^ In the first, rapid phase, 70%–80% of duplicates are lost within ∼60 million years (MY) through large-scale chromosomal rearrangements and deletions. The second, prolonged phase involves the gradual accumulation of mutations in the remaining pairs, resulting in slow gene loss and functional divergence. Consequently, prolonged redundancy during the second phase constitutes a critical period for sub- and neo-functionalization. Teleosts thus provide a powerful model for tracing the long-term trajectory of duplicate genes. While several studies have examined the evolution of duplicated genes following WGD,^22^^,^^25^^,^^26^^,^^27^ relatively few have integrated molecular evolution, expression patterns, and transcriptional regulation across multiple lineages in a unified framework.

Olfactory marker protein (OMP) is strongly and specifically expressed in all mature olfactory sensory neurons (OSNs) in mammals, making it a widely used cellular marker.^28^^,^^29^^,^^30^ Despite its long history as a marker, the molecular function of OMP remained unclear for decades. Recent studies, however, have revealed roles for OMP in calcium homeostasis,^31^ in regulating OSN axonal projection to the olfactory bulb,^32^ and in controlling olfactory adaptation through buffering of the second messenger cAMP.^33^ In teleosts, the OMP gene underwent duplication via the 3 R-WGD, giving rise to *ompa* (OMP2) and *ompb* (OMP1). In our previous study,^34^ we showed that *ompa* and *ompb* exhibit spatially distinct expression patterns in zebrafish, consistent with sub-functionalization. Specifically, *ompa* is expressed in OSNs on the apical side of the olfactory lamella, whereas *ompb* is restricted to basal OSNs, resulting in largely non-overlapping neuronal populations. In that study, comparison with the spotted gar, which possesses a single *omp* gene, suggested that this partitioning reflects sub-functionalization of ancestral expression domains. However, the extent to which this pattern is conserved across teleosts and other actinopterygians remained unclear. Therefore, in the present study, we sought to further investigate the evolutionary trajectories of *omp* genes across multiple lineages, and to extend this analysis to other components of the olfactory signal transduction cascade.

However, previous studies were limited in taxonomic sampling—both in genome surveys and in comparative expression analyses—and lacked comprehensive data from non-teleost actinopterygians. Although some representative non-teleost species, such as the spotted gar, have been investigated, comparative analyses integrating genomic, expression, and regulatory data across multiple lineages remain limited. As a result, the comprehensive evolutionary trajectory of the *omp* gene remains unresolved. In this study, we expanded the taxonomic breadth of genome mining and spatial expression analyses, and combined these with promoter activity assays to provide a higher-resolution reconstruction of post-WGD evolution of *omp* genes. We show that the duplicated *omp* genes accumulated mutations over extended evolutionary periods and diversified into lineage-specific functionalized states. Furthermore, evolutionary analyses of multiple components of the olfactory signal transduction cascade revealed long-term retention of duplicated gene pairs, suggesting the presence of gene dosage constraints in this pathway.

## Results

### Non-functionalization of omp genes after WGD

**Figure 1:**
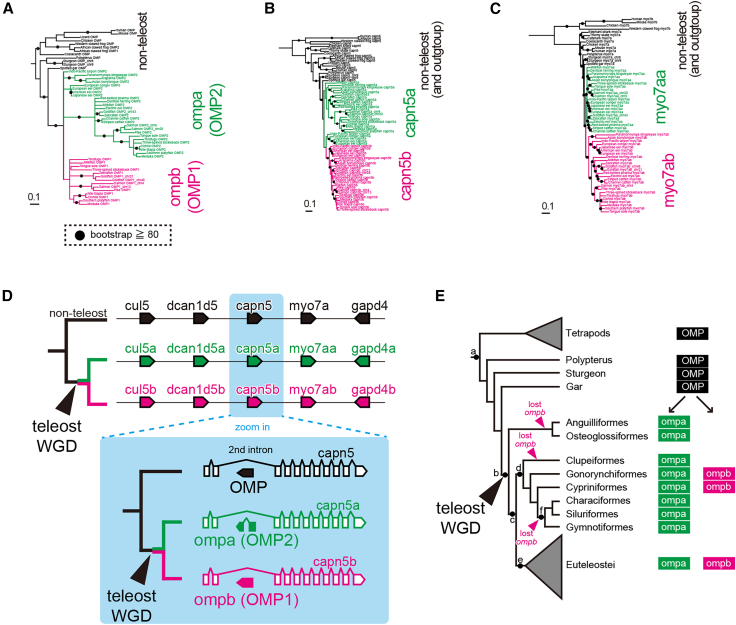
Molecular evolution of the *omp* gene following teleost whole-genome duplication (WGD) (A–C) Maximum-likelihood (ML) phylogenetic trees of (A) *omp*, (B) *capn5*, and (C) *myo7a* genes in vertebrates (detailed phylogenetic trees are shown in Figure S1). Bootstrap values were calculated from 100 replicates; nodes supported by ≥80% bootstrap are marked with black circles. Nodes with <50% support are collapsed into multifurcations in (A) and (C), but are not collapsed in (B). (D) Genomic synteny and schematic representation of the *omp* gene and its neighboring genes (detailed synteny information is provided in Figure S2). The *omp* gene is a nested gene located in the reverse orientation within the second intron of *capn5*. The genomic synteny of *omp* and its neighboring genes (*cul5*, *dcan1d5*, *capn5*, *myo7a*, and *gapd4*) is highly conserved across vertebrates. In teleosts, two sets of these genes are located on different chromosomes, consistent with their origin as WGD-derived duplicates. Notably, in all teleost species examined, the *ompa* gene contains a single intron that divides the coding region. (E) Schematic representation of the molecular evolution of *omp* genes. In non-teleost actinopterygians (e.g., *Polypterus*, sturgeon, and gar), *omp* is present as a single-copy gene (black). Following the teleost WGD, *omp* duplicated into *ompa* (green) and *ompb* (magenta). While *ompa* was retained in all teleost lineages analyzed, *ompb* was secondarily lost in multiple lineages (indicated by magenta arrowheads). Filled circles indicate selected nodes in the phylogeny, and their approximate divergence times (Mya) are as follows: a, ca. 450; b, ca. 300 (teleost-specific WGD); c, ca. 245; d, ca. 220; e, ca. 220; and f, ca. 150. These estimates are based on previously reported divergence times.^24^^,^^35^^,^^36^^,^^37^^,^^38^^,^^39^

First, we retrieved *omp* gene sequences from whole-genome assemblies of a broad range of representative vertebrates (Table S1). Phylogenetic analyses indicated that teleost *omp* genes were separated into two major clades *ompa* and *ompb*, with moderate bootstrap support for *ompb* (64; Figures 1A and S1A). To further validate the classification of *omp* genes, we also analyzed the phylogenies of the *capn5* and *myo7a* genes, as *omp* is nested within the second intron of *capn5*,^34^ and *myo7a* is located adjacent to *capn5*. Similar to *omp*, *capn5*, and *myo7a* genes were divided into *capn5a*/*capn5b* and *myo7aa*/*myo7ab*, respectively, with strong bootstrap support (99 and 100) for the monophyly of both *myo7aa* and *myo7ab* (Figures 1B, 1C, S1B, and S1C). We also compared the genomic synteny surrounding *omp* genes (Figure 1D; details in Figure S2). In teleosts as in other *Osteichthyans*, as previously reported,^34^ *omp* genes are located in an antisense orientation within the second intron of *capn5*, while omp is not present in cartilaginous fishes. We also surveyed available cyclostome genomes (e.g., lamprey) but did not identify clear homologs of *capn5* or *omp*. In particular, no significant hits for *omp* were detected. Genomic synteny around *omp* was well conserved across analyzed species. Non-teleosts generally retained a single-copy synteny set, whereas teleosts maintained two sets on different chromosomes (Figures 1D and S2). As an exception, species that have experienced lineage-specific WGD events—such as *Xenopus laevis*,^40^ sturgeon,^41^ goldfish,^42^ and salmon^43^—show increased copy numbers of *omp* genes. Notably, in goldfish, one copy of *ompa* appears to have been secondarily lost, resulting in a total of three *omp* genes. The presence of two synteny sets in teleosts is characteristic of the teleost WGD and is consistent with our previous study suggesting WGD-mediated duplication of *omp* genes.^34^ Interestingly, some teleost lineages have lost the *ompb* gene, even though their host *capn5* genes are highly conserved. In addition to phylogenetic and synteny analyses, structural features—such as the conversion of *ompa* into a two-exonic gene by intron insertion—indicate that *ompa* has been retained in all examined species, whereas *ompb* has independently become pseudogenized in several lineages (summarized in Figure 1E). Supporting this conclusion, fragments of pseudogenized *ompb* were detected in the second intron of *capn5b* in herring and piranha (Figure S3), suggesting that *ompb* was repeatedly lost through lineage-specific accumulation of mutations, namely non-functionalization. In *Cypriniformes* (e.g., zebrafish and carp), the full-length *ompb* sequence is preserved, whereas in *Characiformes*, *Siluriformes*, and *Gymnotiformes*, *ompb* has become pseudogenized. Because *Cypriniformes* and *Characiformes* diverged approximately 100 MY after the teleost WGD,^35^ this pattern indicates that *ompb* genes survived the initial rapid-loss phase (∼60 MY) and subsequently experienced lineage-specific loss. Taken together, these results demonstrate that although *omp* genes were duplicated in the teleost WGD, *ompb* has been differentially retained or lost across lineages through lineage-specific non-functionalization. Despite these changes, cAMP-binding motifs—predicted to mediate the buffering function of OMP^33^^,^^44^—are highly conserved in both *ompa* and *ompb* across all species analyzed, except for a single amino acid substitution in stickleback *ompb* (Figure S4). This conservation suggests that the cAMP-buffering capacity of the duplicated genes has been largely maintained after WGD.

### Effects of WGD and pseudogenization on spatial expression patterns

**Figure 2:**
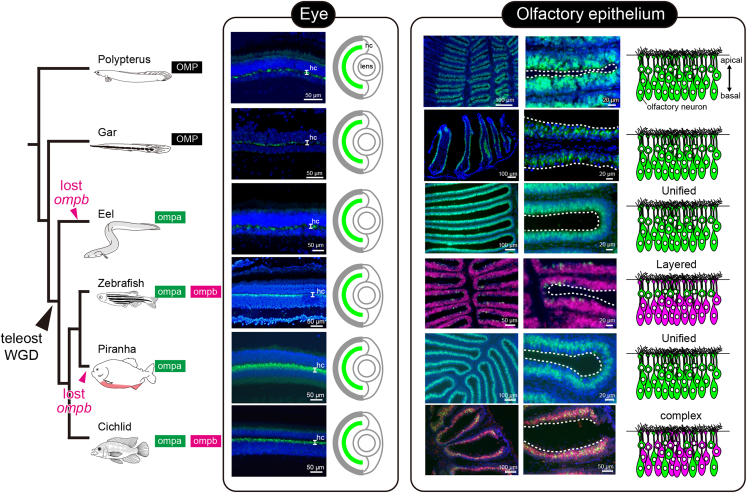
Evolution of the spatial expression patterns of *omp* genes at the cellular level The phylogenetic tree on the left illustrates the evolutionary relationships of the six species analyzed in this study, along with the *omp* genes they possess and an overview of their evolutionary history. The central panels show expression in the eye, particularly in horizontal cells (hc) of the retina, along with a schematic representation. The right images depict expression in the olfactory epithelium and its corresponding schematic. In retina, green fluorescence observed in photoreceptor cells is considered background. Therefore, *in situ* hybridization in the eye was performed using red fluorescence (DyLight-594), and images were converted to green pseudocolor for visualization (only in Figure 2, not in Figure S5). Scale bars: 50 μm in retinal sections; 20, 50, and 100 μm in olfactory epithelium sections, as indicated in each image.

To infer how WGD-driven duplication and subsequent pseudogenization affected *omp* gene expression, we performed fluorescent *in situ* hybridization to visualize spatial expression patterns in six representative fish species (Figure 2). These species were grouped into three categories: (1) non-WGD species (Senegal bichir *Polypterus senegalus* and spotted gar *Lepisosteus oculatus*), (2) teleosts retaining both *omp* genes after WGD (zebrafish *Danio rerio* and African cichlid *Haplochromis sauvagei*), and (3) teleosts that lost *ompb* after WGD (Japanese eel *Anguilla japonica* and red-bellied piranha *Pygocentrus nattereri*). For each category, two phylogenetically distant species were analyzed. We first examined expression in retinal horizontal cells (hc). Consistent with previous studies in zebrafish,^34^ gene expression was detected in the horizontal cells of all analyzed species. In non-teleosts, the single-copy *omp* gene was expressed, whereas in teleosts, which have two copies due to 3 R-WGD, only the *ompa* gene was expressed in horizontal cells. These results suggest that expression in horizontal cells was retained in *ompa*, consistent with sub-functionalization after WGD. In contrast, *capn5*, the host gene of *omp*, was expressed not in horizontal cells but predominantly in the inner nuclear layer (Figure S5).

Next, we examined expression in the olfactory epithelium (OE) (Figure 2). In non-teleosts (*Polypterus* and spotted gar), the single-copy *omp* gene was broadly expressed across the OE, similar to mammals. In teleosts retaining both *ompa* and *ompb* (zebrafish and cichlid), both genes were expressed in the OE. In zebrafish, as previously reported, *ompa* and *ompb* exhibited largely mutually exclusive expression, with *ompa* localized to the apical side of the olfactory lamellae and *ompb* to the basal side; the *ompb*-expressing cells were predominant. In cichlid, *ompa* and *ompb* were also expressed mutually exclusively, with *ompb*-expressing cells predominating, although a clear layered expression pattern was not observed as in zebrafish. In teleosts that lost *ompb* (eel and piranha), *ompa* was expressed across the entire population of OSNs. These results suggest that each fish species express its available set of *omp* gene(s) across the OSN population in a species-specific manner.

### Evaluation of promoter activity across omp genes

**Figure 3:**
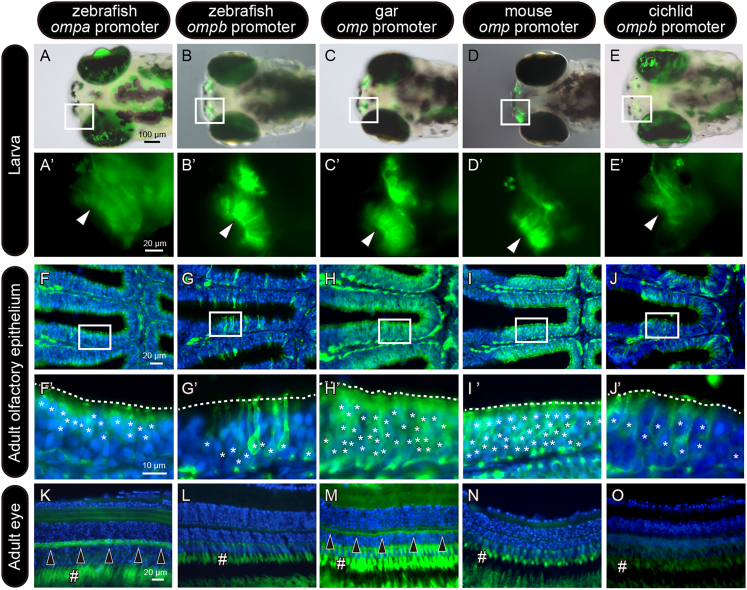
Reporter assay of *omp* promoter activity using transgenic zebrafish lines (A–E) Reporter fluorescence in 5 dpf (days post-fertilization) larvae of transgenic zebrafish. Each panel shows an overlay of bright-field and fluorescent images. (A’–E’) High-magnification images of the olfactory organ in the corresponding transgenic zebrafish larvae shown in (A–E). (F–J) Sections of the olfactory epithelium in adult transgenic zebrafish lines. The boxed regions are shown at higher magnification in (F’–J’). (K–O) Sections of the eye in adult transgenic zebrafish lines. The promoters of zebrafish *ompa* and gar *omp* drove reporter expression in horizontal cells of the retina (arrowheads). Green fluorescence observed in photoreceptor layers, marked by (#), is considered background signal rather than specific reporter expression. Scale bars: 100 μm in (A–E); 20 μm in (A’–E’) and (F–J); 10 μm in (F’–J’); and 20 μm in (K–O).

**Figure 4:**
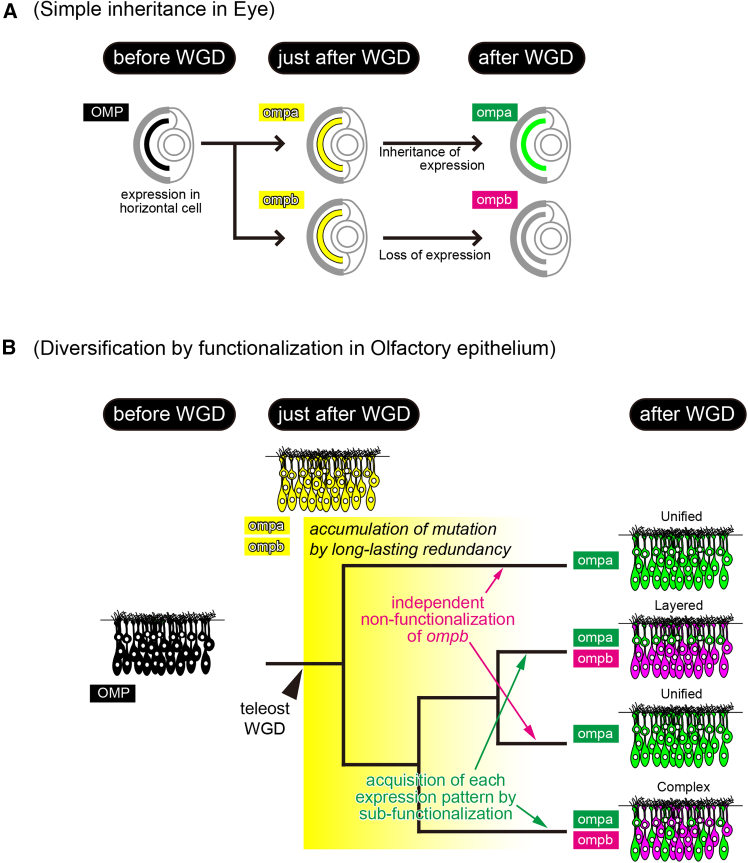
Schematic representation of the impact of teleost WGD on the spatial expression patterns of *omp* genes (A) Evolutionary changes in *omp* expression in horizontal cells of the eye—functionalization by simple inheritance of the original expression pattern in one of the duplicated genes. Before the teleost WGD, *omp* was expressed in retinal horizontal cells (before WGD). Immediately after the WGD, both *ompa* and *ompb* were likely expressed in horizontal cells (just after WGD). Over the long course of evolution, *ompa* retained expression in horizontal cells, whereas *ompb* lost this expression (after WGD). (B) Evolutionary changes in *omp* expression in OSNs—diversification of expression patterns through accumulation of mutations under long-lasting redundancy. Prior to the teleost WGD, *omp* was expressed throughout mature OSNs (before WGD). Immediately after WGD, both *ompa* and *ompb* were likely expressed throughout mature olfactory neurons (just after WGD). Over evolutionary time, the redundancy maintained by the two pairs of genes (*ompa* and *ompb*) allowed accumulation of *cis*-regulatory mutations, leading to diversification of expression patterns during species divergence (after WGD).

To investigate the mechanisms underlying the diversification of spatial expression patterns among *omp* genes, we generated zebrafish reporter lines driven by promoter sequences from various species (Figure 3). All reporter lines exhibited clear fluorescent signals in the olfactory organ at 5 days post fertilization (Figures 3A–3E and 3A’–3E’). In adult OE, the zebrafish *ompa* promoter drove Venus expression in the apical layer (Figures 3F and 3F’), whereas the *ompb* promoter drove expression in the basal layer of the olfactory lamellae (Figures 3G and 3G’). These expression layers closely mirrored the endogenous mRNA patterns, indicating that each promoter accurately captures the transcriptional properties of its corresponding *omp* gene. In contrast, promoter sequences from species that did not experience the teleost WGD—the spotted gar (Figures 3H and 3H’) and mouse (Figures 3I and 3I’)—drove Venus expression across both layers, even in a different species (zebrafish OE). Likewise, promoters derived from cichlid *omp* genes produced complex expression patterns (Figures 3J and 3J’) that mirrored the original cichlid OE patterns, rather than the layered organization characteristic of zebrafish *ompa* and *ompb*. Similarly, in the adult retina, Venus expression driven by each promoter consistently reflected the endogenous expression layers of the corresponding genes (Figures 3K–3O). Taken together, these results indicate that *omp* promoters from diverse species can reproduce their species-specific expression patterns even in a different species (zebrafish) cellular environment. This pattern strongly supports the view that the diversification of *omp* expression among teleosts has been shaped primarily by mutations accumulated within *cis*-regulatory elements, rather than by shifts in *trans*-regulatory factors, chromatin accessibility, or the spatial arrangement of OSN subtypes. A summary of the molecular evolutionary patterns revealed in this study is provided in Figure 4.

To further examine the *cis*-regulatory architecture of omp genes, we performed comparative sequence analysis of upstream regions using VISTA plots (Figure S6). A conserved non-coding sequence was identified upstream of *ompa* across multiple species, whereas no comparable conserved region was detected in the upstream region of *ompb*. Notably, this conserved region is included within the promoter fragments used in our reporter assays. However, the functional relevance of this sequence remains unclear, and it is currently unknown whether it contributes to the regulation of expression in the OE, retina, or other tissues.

### Dosage constraints and long-lasting redundancy in olfactory signaling cascade

**Figure 5:**
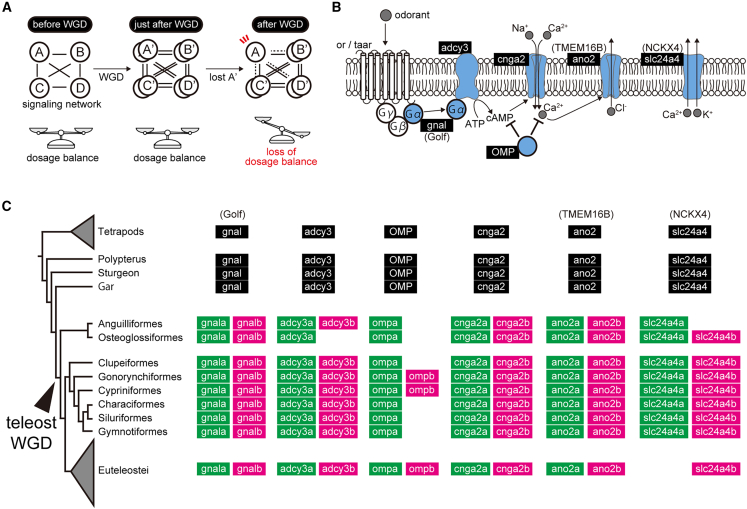
Dosage constraints hypothesis in the olfactory signaling cascade following teleost WGD (A) Schematic illustration of the dosage constraints hypothesis. At the cellular level, genes constituting a signaling network or metabolic pathway maintain appropriate relative levels of translated proteins (before WGD). WGD doubles all genes, thereby proportionally increasing the amount of each protein while maintaining their relative balance (just after WGD). Subsequently, if one of the duplicated genes in the network becomes pseudogenized, the stoichiometric balance of protein components is disrupted, leading to network collapse (after WGD). Because individuals with a disrupted network are eliminated by purifying selection, the WGD-derived gene pairs tend to be retained over evolutionary time—a process explained by the dosage constraints hypothesis. (B) Schematic diagram of a representative vertebrate olfactory signaling cascade. (C) Molecular evolution of olfactory signaling cascade genes before and after teleost WGD, showing the presence or absence of each gene across lineages, based on phylogenetic and genomic synteny analyses (see also Figure S6).

Inoue et al. estimated that approximately 72%–82% of WGD-derived duplicate pairs experienced loss of one paralog within the first ∼60 MY.^24^ To clarify how *omp* genes escaped this early phase of extensive gene loss, we analyzed the molecular evolutionary patterns of genes involved in the olfactory signal transduction cascade (Figure 5). Unlike small-scale duplications, WGD duplicates entire interacting gene networks. As a result, overall dosage balance is preserved even as copy numbers increase. In a dosage-sensitive network, the loss of even a subset of components disrupts functional stoichiometry, thereby constraining the loss of duplicate genes (Figure 5A; the dosage-constraints hypothesis, also referred to as dosage-balance or dosage-compensation).^45^^,^^46^^,^^47^ The vertebrate olfactory signal transduction cascade—particularly well characterized in mouse—includes the genes *gnal*, *adcy3*, *omp*, *cnga2*, *ano2*, and *slc24a4* (Figure 5B).^48^^,^^49^^,^^50^^,^^51^^,^^52^ Using whole-genome searches, phylogenetic analyses, and genomic synteny comparisons, we identified the orthologs and WGD-derived paralogs of these six genes across teleost lineages (Figure S7). Strikingly, in many teleost clades, both WGD-derived pairs of all six genes have been retained (Figure 5C), and in most cases, these duplicates form clearly separated paralogous clades (a and b). However, some exceptions were observed. For *gnal*, the use of *gnas* as an outgroup resulted in an unexpected topology in which non-teleost sequences were placed within the teleost clade, likely due to the large evolutionary distance between these genes. To address this matter, we examined both outgroup-rooted and unrooted trees, and in both cases, the duplicated *gnal* genes were consistently resolved into two distinct clades (Figure S7). For *adcy3*, the sarcopterygian topology showed slight deviations from the expected species relationships; however, the relevant nodes were supported by low bootstrap values, suggesting limited phylogenetic signal. Because *adcy8* represented the closest identifiable paralogous gene family, it was retained as the outgroup for the analysis. In addition, for *ano2*, sequences from *Elopomorpha* and Osteoglossomorpha did not clearly separate into a and b clades, possibly reflecting long-branch attraction or lineage-specific rate variation. Together, these results indicate that, in contrast to the genome-wide trend reported in previous studies,^24^ olfactory signaling genes exhibit exceptional long-term retention of WGD-derived paralogs, supporting the idea that dosage constraints contributed to the prolonged preservation of this signaling network.

## Discussion

Gene duplication is a major driver of molecular evolution, and, in particular, WGD, which duplicates the entire gene repertoire, represents a prominent example.^1^ While WGD can facilitate the acquisition of novel traits and diversification of morphology and function,^14^^,^^21^^,^^22^ only a small subset of duplicated genes actually contributes to these innovations. The majority of duplicated gene pairs are thought to rapidly revert to a single-copy state due to functional redundancy.^23^^,^^24^ Nevertheless, many gene pairs retain long-term redundancy and undergo lineage-specific functional divergence, including vertebrate Gnrh1/Gnrh3,^26^ teleost cholecystokinin a/b,^53^^,^^54^ yeast ribosomal protein genes,^55^ and plant SEP1/SEP2 and SHP1/SHP2.^56^^,^^57^ These cases illustrate that WGD-derived genes do not necessarily undergo rapid divergence or loss but can follow diverse evolutionary trajectories through prolonged redundancy. Here, we use the term “redundancy” to refer to the presence of duplicated genes that have not yet undergone substantial functional divergence, rather than implying complete functional interchangeability or dispensability of either copy.

Before discussing the evolutionary trajectories of *omp* genes, it is important to consider the origin of the *omp*-containing genomic regions. Although the conserved synteny observed across teleost species is consistent with a teleost-specific 3 R origin, the relatively small size of the genomic block and extensive post-3R chromosomal rearrangements—such as translocations, fusions, and differential gene loss—can obscure the original correspondence between duplicated chromosomal regions.^36^^,^^58^ To address this, we expanded our synteny analysis to include medaka, in which duplicated chromosomal correspondence is more clearly preserved.^36^^,^^58^ In medaka, *ompa* and *ompb* are located on paralogous chromosomal regions (Figure S2: chr. 13 and 14), supporting their origin from the teleost-specific WGD. In contrast, in species such as zebrafish, extensive genomic rearrangements likely obscure the ancestral chromosomal relationships. Consistent with this interpretation, species that have undergone independent lineage-specific WGD events, such as goldfish, salmon, sturgeon, and *Xenopus laevis* also possess duplicated *omp* gene copies, although goldfish *ompa* have been secondarily lost. Taken together, while the current chromosomal configuration should be interpreted with caution, the conserved gene content, cross-species synteny, and consistent duplication patterns support the interpretation that the *omp*-containing region originated from the teleost 3 R event.

Teleosts, which experienced WGD approximately 300 MYA, provide an ideal model to investigate the long-term molecular evolution of duplicated genes.^23^^,^^24^ However, studies that integrate multiple aspects—such as gene sequence, expression patterns, and transcriptional regulation—to trace the long-term evolutionary trajectory following WGD remain limited. Here, we focused on the teleost WGD-derived *omp* (OMP) genes to explore diverse trajectories of gene functional divergence over ∼300 MY. By combining genomic analyses, comparative expression studies, and promoter activity assays, we showed that duplicated *omp* gene pairs have undergone lineage-specific accumulation of mutations, resulting in either sub-functionalization or non-functionalization. As illustrated in Figure 4, *ompa* and *ompb* likely shared ancestral expression patterns immediately after WGD. Subsequently, expression in the visual system was largely and directly inherited by *ompa*, whereas in the olfactory system, lineage-specific mutations led to diverse expression patterns, resulting in sub-functionalization or non-functionalization. These results suggest that functionalization in the olfactory system was not a simple inheritance of ancestral function but rather the outcome of a complex and dynamic molecular evolutionary process.

In addition to the teleost-specific 3 R event, lineage-specific genome duplication events have also contributed to gene expansion in other vertebrate lineages. For example, *X*. *laevis* has undergone allotetraploidization,^40^ resulting in duplicated gene sets, including *omp*. Previous studies have reported that the duplicated *omp* genes in *X. laevis* show largely overlapping expression patterns with only minor differences, suggesting limited divergence following duplication.^59^ Given that the allotetraploidization event in *X. laevis* is relatively recent (∼30 MYA), this pattern may reflect an early stage of functional divergence. In contrast, the duplicated *omp* genes analyzed in this study, which originated from the teleost WGD, exhibit clear transcriptional differentiation. This difference in evolutionary timescale may underlie the extent of sub-functionalization observed across lineages. Similarly, teleost lineages that experienced additional genome duplication events, such as the goldfish 4 R event^42^ and salmonid^43^-specific duplications, also retain duplicated copies of these genes, although some copies have been secondarily lost. Further comparative analyses of expression patterns among duplicated *omp* genes across lineages with independent genome duplication events (e.g., *X. laevis*, sturgeon, goldfish, and salmon) will help clarify how redundancy is maintained and resolved over evolutionary time.

### Functional role and evolutionary origin of omp

Our study revealed that WGD-derived *ompa*/*ompb* pairs have been largely possessed retained in many teleosts, and the two genes showed clear transcriptional differentiation in zebrafish and cichlid (Figure 2). Moreover, *ompa* expression in horizontal cells of the retina is conserved in teleosts, and similar expression is observed in non-teleostean actinopterygii (*Polypterus* and gar) that possess a single-copy *omp* gene. These patterns indicate sub-functionalization, although physiological divergence between ompa and ompb remains unclear. Previous studies have shown that the cAMP-binding motif of the omp protein is highly conserved from mammals to fish.^33^^,^^44^ In our analysis distinguishing between *ompa* and *ompb*, this motif was highly conserved in both, preventing functional differentiation from being inferred based on sequence alone (Figure S4).

The *omp* is found in both bony fishes and tetrapods, and its origin has been suggested to date back to at least the common ancestor of bony fishes.^34^ In this study, we confirmed that *omp* is absent from the in cartilaginous fishes, based on comprehensive genome-wide searches, including but not limited to the intronic regions of *capn5*, strongly supporting that *omp* was newly acquired in the bony fish lineage (Figure S2). Furthermore, *omp* expression in horizontal cells suggests that this trait predates the common ancestor of ray-finned fishes (Figure 2). Expression of *omp* in the retina has not been well characterized in birds and amphibians, and therefore the evolutionary timing of horizontal cell expression remains unresolved. However, *omp* expression in the retina is not detected in mouse,^34^ and data from basal sarcopterygians such as lungfish are limited, leaving the precise timing of acquisition of horizontal cell expression unresolved. We also surveyed available cyclostome genomes (e.g., lamprey) but did not identify clear homologs of *capn5* or *omp*. In particular, no significant hits for *omp* were detected. While this may suggest that *omp* originated after the divergence of jawless vertebrates, incomplete genome assemblies and annotation limitations should be considered. Given that jawless vertebrates are known to possess horizontal cells,^60^^,^^61^^,^^62^ it is likely that *omp* function in horizontal cells evolved independently of the acquisition of horizontal cells during vertebrate evolution.

The apparent restriction of *omp* expression in horizontal cells to ray-finned fishes may reflect visual adaptations to aquatic environments. In aquatic environments, turbidity reduces light transmission and shifts spectral composition dynamically, making rapid and flexible light adaptation essential.^63^^,^^64^ Horizontal cells in teleosts are strongly modulated by dopamine via the D1 receptor-cAMP-PKA pathway, which regulates gap-junction coupling and light responsiveness.^65^^,^^66^ Because *omp* buffers intracellular cAMP levels to regulate sensory adaptation in olfactory neurons,^33^ we hypothesize that *ompa* in horizontal cells may similarly stabilize cAMP dynamics to fine-tune light-adaptation kinetics. Such modulation could help maintain visual performance under dynamic underwater light environments. Future knockout studies will be needed to determine its precise physiological function.

### Regulation of omp gene expression

The *omp* gene is a nested gene located within an intron of the *capn5* host gene (Figure 1D). The regulatory relationship between host and nested genes—whether they are co-expressed, mutually exclusive, or independent—has been debated for decades.^67^^,^^68^^,^^69^ Previous studies in mouse showed that in OSNs where the nested *omp* gene is expressed, the host gene *capn5* is not expressed.^70^ In the present study, analysis of *capn5* expression in the retina of gar and zebrafish revealed expression in the inner nuclear layer, whereas no signal was detected in horizontal cells expressing *omp* (Figure S5). This separation of expression suggests that the *omp* gene is regulated independently of its host gene. In mammals, short promoter regions of approximately 0.3 kb are sufficient to drive mature OSN-specific expression,^71^^,^^72^^,^^73^ and in the present study, upstream sequences of ∼1 kb mimicked endogenous expression patterns. These observations indicate that *omp* transcription is driven by a short upstream sequence across vertebrates and is regulated independently of the host gene. In this context, our discussion of regulatory redundancy is primarily based on proximal regulatory elements captured by these upstream sequences. We acknowledge that distal regulatory elements and higher-order chromatin organization may not be fully preserved following duplication. However, the ability of short promoter regions to recapitulate endogenous expression patterns suggests that, at least at the level of proximal regulation, duplicated *omp* genes retain a substantial degree of functional redundancy. Therefore, our interpretation of regulatory redundancy is limited to proximal mechanisms and does not necessarily extend to genome-wide regulatory architecture. Although analysis of the cichlid *ompa* promoter would further strengthen the comparative framework, technical limitations prevented its inclusion in the present study. Nevertheless, our conclusions are supported by consistent functional and expression patterns observed for *ompb* across species, indicating that the key evolutionary trends described here are robust.

### Dosage constraints promote maintenance and functional divergence of teleost olfactory genes

An important question in molecular evolution concerns why some WGD-derived gene pairs, such as *ompa*/*ompb*, have been retained over long evolutionary periods. In this study, we hypothesized that dosage constraints within OSNs have promoted the maintenance of *ompa*/*ompb* gene pairs. According to the dosage-constraints hypothesis,^45^^,^^46^^,^^47^ maintaining the quantitative balance of component genes within molecular complexes functioning in the cell is advantageous under natural selection. Consistent with this hypothesis, Sato et al. reported that gene pairs retained long-term after the teleost-WGD tend to have numerous interacting partners.^23^ In this study, we analyzed multiple genes comprising the olfactory transduction cascade (*gnal*, *adcy3*, *cnga2*, *ano2*/*tmem16b*, and *slc24a4b*) and found that all duplicated gene pairs are retained in teleosts (Figure 5). This retention is remarkable considering that 72%–82% of gene pairs were lost during the first ∼60 MY following the teleost-WGD^24^ and that the retention rate of WGD-derived gene pairs in zebrafish is only ∼26%.^74^ Here, we use the term “redundancy” to refer to the presence of duplicated genes that have not yet undergone substantial functional divergence, rather than implying complete functional interchangeability. These observations imply that strong dosage constraints act on the olfactory pathway, maintaining functional redundancy. Moreover, prolonged maintenance of redundancy facilitates neo- or sub-functionalization.^75^ The partial co-expression of *ompa* and *ompb* in a subset of OSNs (Figure 2)^34^ may reflect this transitional state. Taken together, long-term redundancy after WGD, followed by subsequent functional differentiation, likely promoted diversification of the olfactory transduction cascade and provided an evolutionary foundation for teleosts to adapt to diverse olfactory environments. A more detailed understanding of the divergence and evolutionary trajectories of olfactory signaling molecules in fishes remains an important future goal.

### Limitations of the study

This study focused primarily on the evolutionary retention, expression divergence, and regulatory evolution of duplicated olfactory signaling genes after teleost WGD. Although our results support prolonged redundancy and sub-functionalization of duplicated *omp* genes, the physiological and functional differences between *ompa* and *ompb* remain unclear. In addition, whether the long-term retention of duplicated signaling components directly contributed to diversification of olfactory function or species diversity in teleosts remains to be determined. Future functional and behavioral studies will help clarify the biological consequences of prolonged redundancy in the teleost olfactory system.

## Resource availability

### Lead contact

Further information and requests for resources and reagents should be directed to and will be fulfilled by the lead contact, Tatsuki Nagasawa (nagasawa.t.424e@m.isct.ac.jp).

### Materials availability

Plasmids and transgenic zebrafish lines generated in this study are available from the lead contact upon reasonable request. Transgenic zebrafish lines [Tg(DreOmpa:Venus), Tg(DreOmpb:Venus), Tg(HsaOmp:Venus), Tg(MmuOmp:Venus), and Tg(LocOmp:Venus)] and the corresponding reporter constructs generated in this study are available from the lead contact upon reasonable request.

### Data and code availability

• Data: All sequence data analyzed in this study are listed in Table S1, including the corresponding accession numbers.
• Code: This study did not generate any custom code.
• Additional information: Newly generated transgenic zebrafish lines are available from the lead contact upon reasonable request. Any additional information required to reanalyze the data reported in this paper is available from the lead contact upon request.

## Acknowledgments

We thank Prof. Koichi Kawakami (National Institute of Genetics, Japan) for kindly providing the *Tol2* plasmid. We thank the National BioResource Project (NBRP), Japan, for providing the RIKEN wild-type zebrafish strain used in this study. We thank Mr. Yujiro Kawabe for assistance with scientific illustrations. We thank the Materials Analysis Division, Open Facility Center, Institute of Science Tokyo for technical assistance with DNA sequencing. This study was supported in part by Grant -in -Aid for Young Scientists (10.13039/501100001691JSPS 10.13039/501100001691KAKENHI Grant Number 23K14249), the Sasakawa Scientific Research Grant from the 10.13039/501100007807Japan Science Society to T.N., and Grant in Aid for Scientific Research B (10.13039/501100001691JSPS 10.13039/501100001691KAKENHI Grant 24K02074) to M.N.

## Author contributions

Conceptualization, T.N. and M.N.; methodology, T.N., H.F., T.O., and M.N.; investigation, T.N., H.F., and T.O.; formal analysis, T.N.; writing – original draft, T.N.; writing – review & editing, T.N. and M.N.; supervision, M.N.; funding acquisition, T.N. and M.N.

## Declaration of interests

The authors declare no competing interests.

## Declaration of generative AI and AI-assisted technologies in the writing process

The authors used ChatGPT for language polishing and stylistic refinement of the manuscript. All AI-assisted text was carefully reviewed, revised, and approved by the authors. The authors take full responsibility for the content of the manuscript.

## STAR★Methods

### Key resources table

**Table:** 

REAGENT or RESOURCE	SOURCE	IDENTIFIER
**Antibodies**		

anti-fluorescein-POD Fab fragments	Roche	Cat#11426346910; RRID: AB_840257
anti-digoxigenin-POD Fab fragments	Roche	Cat#11207733910; RRID: AB_514500
Streptavidin-Alexa Fluor 488	Invitrogen	Cat#S11223
anti-digoxigenin antibody DyLight 594	Vector Laboratories	Cat#DI-7594-.5; RRID: AB_2336409

**Chemicals, peptides, and recombinant proteins**		

MS-222	Combi-Blocks	Cat#QC-9764
Paraformaldehyde (PFA)	FUJIFILM Wako	Cat#162-16065
OCT compound	SAKURA	Cat#45833
H_2_O_2_	FUJIFILM Wako	Cat#086-07445
proteinase K	FUJIFILM Wako	Cat#161-28701
Blocking Reagent	Roche	Cat#10057177103
VECTASHIELD with DAPI	Vector Laboratories	Cat#H-1200

**Critical commercial assays**		

mMESSAGE mMACHINE SP6 kit	Invitrogen	Cat#AM1340
DIG RNA labeling mix	Roche	Cat#11277073910
FITC RNA labeling mix	Roche	Cat#11685619910
TSA Biotin System	Akoya Biosciences	Cat#NEL700A001KT
TSA Plus DIG system	Akoya Biosciences	Cat#NEL748001KT

**Experimental models: Organisms/strains**		

*Polypterus senegalus*	Local dealer	N/A
*Lepisosteus oculatus*	Local dealer	N/A
*Anguilla japonica*	Local dealer	N/A
*Pygocentrus nattereri*	Local dealer	N/A
*Haplochromis sauvagei*	Local dealer	N/A
*Danio rerio* (RIKEN Wild type strain)	National BioResource Project (Japan)	https://shigen.nig.ac.jp/zebra/
Generated transgenic zebrafish lines	This study	N/A
*Escherichia coli* strain DH5α	Takara Bio	Cat#9057

**Oligonucleotides**		

See Tables S1, S2, S3	This study	N/A

**Recombinant DNA**		

pT2AL200R150G	Kawakami et al.^76^	N/A
pCS-zTP	Kawakami et al.^76^	N/A
omp promoter:Venus constructs	This study	N/A
pGEM-T vector	Promega	Cat#A1360

**Software and algorithms**		

MAFFT v7	Nakamura et al.^77^	https://mafft.cbrc.jp/alignment/software/
Genewise2	Madeira et al.^78^	https://www.ebi.ac.uk/∼birney/wise2/
ModelTest-NG	Darriba et al.^79^	https://github.com/ddarriba/modeltest
RAxML-NG v1.2.0	Kozlov et al.^80^	https://github.com/amkozlov/raxml-ng
iTOL v6	Letunic and Bork^81^	https://itol.embl.de/
Genomicus	Nguyen et al.^82^	https://www.genomicus.bio.ens.psl.eu/genomicus
NCBI Genome Data Viewer	NCBI	https://www.ncbi.nlm.nih.gov/gdv/
mVISTA	Brudno et al.^83^	http://lagan.stanford.edu/lagan_web/index.shtml

### Experimental model and study participant details

#### Animals and ethical approval

Adult Spotted gar (*Lepisosteus oculatus*), Gray bichir (*Polypterus senegalus*), Japanese eel (*Anguilla japonica*), Red-bellied piranha (*Pygocentrus nattereri*), and African cichlid (*Haplochromis sauvagei*) were obtained from commercial suppliers. Zebrafish (*Danio rerio*) of the inbred RIKEN Wild type strain, provided by the National BioResource Project (NBRP, Japan), were used in this study. All fish were maintained in breeding tanks at 25°C–29°C under a 12 h light/12 h dark photoperiod and fed two to three times daily. For tissue sampling, fish were anesthetized with MS-222 and euthanized by rapid decapitation, after which the olfactory rosettes and eyes were extracted. Genomic DNA for amplification of the mouse promoter sequence was kindly provided by Prof. Junji Hirota (Institute of Science Tokyo). Accordingly, no live mouse experiments were conducted in this study. Adult fish were used in this study. Sex was not recorded and was not considered as an experimental variable.

#### Ethics statement

All animal experiments were conducted in accordance with institutional guidelines and approved by the institutional biosafety and animal care committees of the Institute of Science Tokyo. All recombinant DNA experiments were approved by the institutional biosafety committee of the Institute of Science Tokyo (I2019035 and I2024021).

### Method details

#### Isolation and collection of gene sequences

The identification and isolation of gene sequences were performed following previously published methods^84^ in accordance with slight modifications. Gene sequences were either retrieved from the NCBI database or newly predicted from whole-genome assemblies deposited in NCBI. To identify the target loci, TBLASTN searches were performed against the genomic sequences of each species using amino acid sequences of orthologous genes from closely related species as queries. Subsequently, exon sequences were extracted from the surrounding genomic regions using Genewise2,^78^ with the same orthologous amino acid sequences as references. All accession numbers and the sequences used in this study are summarized in Table S1. We refer to teleost WGD-derived paralogs as ‘a’ and ‘b’ following conserved synteny (e.g., *cnga2a*/*cnga2b*). ANO2 is also known as TMEM16B.

#### Molecular phylogenetic and genomic synteny analysis

Maximum-likelihood phylogenetic analyses were performed using RAxML-NG.^80^ Amino acid sequences of each gene were aligned using MAFFT.^77^ The best-fit substitution models were determined using ModelTest-NG,^79^ and phylogenetic trees were inferred with RAxML-NG with 100 bootstrap replicates. Phylogenetic trees were visualized using iTOL ver. 6^78^ (https://itol.embl.de/). Outgroup sequences were selected based on previously reported gene family classifications and phylogenetic studies.^85^^,^^86^^,^^87^^,^^88^^,^^89^^,^^90^^,^^91^^,^^92^^,^^93^ For each gene, appropriate outgroups were chosen when clear homologous relationships could be established; however, for *omp*, no suitable outgroup gene was identified, and analyses were conducted without an outgroup. Genomic synteny comparisons were performed using Genomicus^82^ (https://www.genomicus.bio.ens.psl.eu/genomicus-110.01/cgi-bin/search.pl) and NCBI Genome Data Viewer^94^ (https://www.ncbi.nlm.nih.gov/gdv/). Homologous relationships among genes were carefully validated through TBLASTN searches against the available genome datasets of each species.

#### Comparative analysis of upstream regulatory sequences

Comparative analysis of upstream sequences of *omp* genes was performed using the VISTA tool (http://lagan.stanford.edu/lagan_web/index.shtml).^83^ Genomic sequences corresponding to regions upstream of *omp* genes were retrieved from publicly available genome assemblies. Sequence alignments were conducted using the mVISTA program with default parameters, using the medaka *ompa* upstream region as a reference. Conserved non-coding sequences were identified based on sequence conservation profiles generated by VISTA.

#### Construction of reporter constructs and establishment of transgenic zebrafish lines

For the establishment of each transgenic zebrafish line, we used the plasmids pT2AL200R150G and pCS-zTP, which were kindly provided by Prof. Koichi Kawakami (National Institute of Genetics) (https://ztrap.nig.ac.jp/trans.html), following the procedures described previously^76^ with minor modifications. The GFP sequence of pT2AL200R150G was replaced with the Venus fluorescent protein sequence (hereafter referred to as the Venus vector), which was used as the backbone for all reporter constructs. Genomic DNA was used as a template to amplify the upstream region of the *omp* gene by PCR. The primers used for amplification are listed in Table S2. Because PCR amplification of the upstream region of zebrafish *ompa* was difficult due to repetitive sequences, a DNA fragment excluding the repetitive region was synthesized commercially (Integrated DNA Technologies; the sequence is shown in Figure S8). The amplified fragments were cloned into the Venus vector, and plasmid DNA was obtained after transformation into *Escherichia coli*. The resulting plasmid and transposase mRNA, synthesized from pCS-zTP using SP6 RNA polymerase (mMESSAGE mMACHINE SP6 Transcription Kit; Invitrogen), were co-injected into zebrafish fertilized eggs. Individuals showing fluorescence signals in OSNs at 5 days post fertilization (dpf) were raised as founder (F0) fish. The matured F0 fish were crossed with wild-type individuals, and the offspring showing similar fluorescence signals were selected and maintained as F1. The transgenic lines showing stable fluorescence expression over F2 and subsequent generations were established and used for analyses.

#### Preparation of frozen sections and *in situ* hybridization

Frozen sections were prepared as follows. Tissues were dissected in chilled 4% paraformaldehyde (PFA) and subsequently fixed overnight at 4°C. For the eyes, the cornea was carefully punctured with forceps to facilitate the penetration of PFA. After fixation, the samples were thoroughly rinsed in PBS, cryoprotected overnight in 30% sucrose solution, and embedded in OCT compound (SAKURA Finetek Japan). Embedded tissues were sectioned at a thickness of 10 μm using a cryostat, mounted onto glass slides, and either observed under fluorescence microscopy after embedding with VECTASHIELD mounting medium with DAPI (Vector Laboratories) or stored at −80°C until use for *in situ* hybridization. In order to visualize mRNA localization, *in situ* hybridization was performed according to previous studies^34^^,^^95^ with slight modification. Specific primers (listed in Table S3) were designed within the coding region of the *omp* genes, and PCR amplification was performed using OE cDNA as a template. Amplified fragments were cloned into the pGEM-T vector (Promega). The plasmids were linearized by restriction enzyme digestion, and DIG-labeled RNA probes were synthesized using the DIG RNA Labeling Kit (Roche). For dual *in situ* hybridization, FITC-labeled RNA probes were synthesized in the same manner using the FITC RNA Labeling Mix (Roche). For single-color detection, prior to hybridization, the sections were treated with proteinase K for protein digestion, followed by post-fixation, inactivation of endogenous peroxidase (POD) and alkaline phosphatase (AP), and acetylation of amino groups. Hybridization was carried out overnight at 55°C–70°C. After stringent washing to remove unbound probes, sections were incubated with 1% Blocking Reagent (Roche), followed by anti-digoxigenin-POD Fab fragments (Roche) at 4°C overnight. Signals were amplified using the TSA Biotin System (Akoya Biosciences), and after blocking endogenous biotin and streptavidin, visualization was performed with Streptavidin-Alexa Fluor 488 conjugate (Invitrogen). Sections were mounted in VECTASHIELD with DAPI for nuclear counterstaining and imaging. For dual-color detection, the procedures were generally identical to those for single-color detection, except that signal amplification was performed using the TSA Plus DIG System (Akoya Biosciences), followed by POD inactivation with H_2_O_2_ treatment. Sections were then reacted with anti-fluorescein-POD Fab fragments (Roche) and anti-digoxigenin antibody DyLight 594 (Vector Laboratories), and further amplified using the TSA Biotin System. Visualization was achieved with Streptavidin-Alexa Fluor 488 (Invitrogen), and the sections were mounted and observed as described above. In the zebrafish retina, green fluorescence is frequently observed in photoreceptor cells as background signal. To avoid this issue, *in situ* hybridization in the eye was performed using red fluorescence (DyLight-594), and the resulting images were subsequently converted to green pseudocolor for visualization.

### Quantification and statistical analysis

Phylogenetic confidence was assessed by bootstrap analysis with 100 replicates using RAxML-NG. No additional statistical hypothesis testing was performed in this study. Details regarding sample numbers (n) and experimental observations are provided in the corresponding figure legends where applicable.

## Supplemental information

Document S1. Figures S1–S8

Table S1. Accession numbers and sequences used in this study

Table S2. Primer sequences used for reporter construct generation

Table S3. Primer sequences used for *in situ* hybridization probe design
