# Supplementary material for: Evolutionary trajectories of teleost olfactory signaling genes shaped by long-term redundancy after whole-genome duplication
Source: iScience. 2026 Jun 30;29(7):116564. doi: 10.1016/j.isci.2026.116564 (PMC13378369; doi:10.1016/j.isci.2026.116564)
Supplement: Document S1. Figures S1–S8 [file mmc1.pdf]

iScience, Volume *29*

Supplemental information

Evolutionary trajectories of teleost olfactory signaling genes shaped by long-term redundancy after whole-genome duplication

Tatsuki Nagasawa, Hanami Fujisaki, Takahiro Ogo, and Masato Nikaido

A

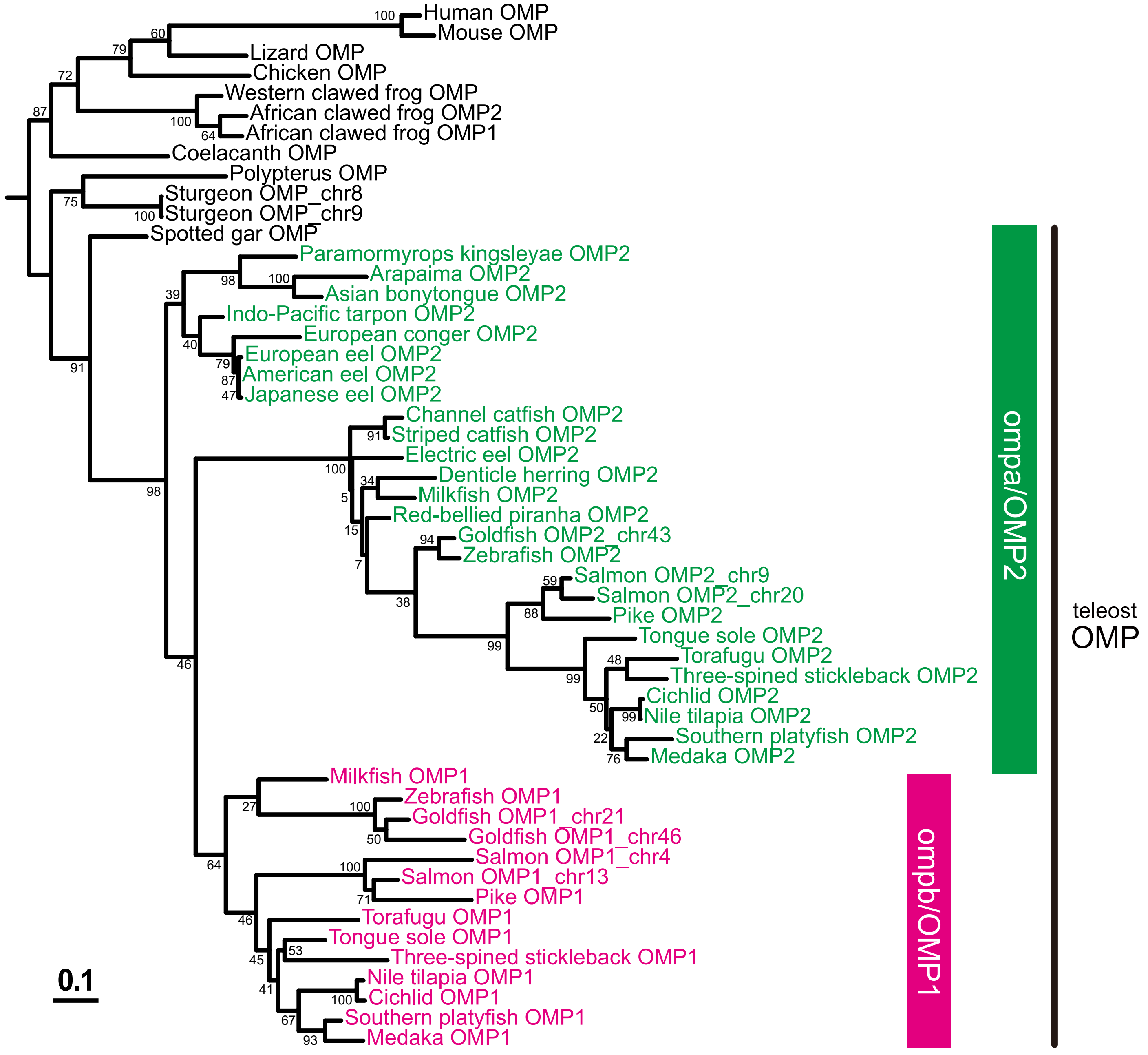

Figure S1A. Detailed maximum-likelihood phylogenetic tree of *omp* genes shown in Figure 1.

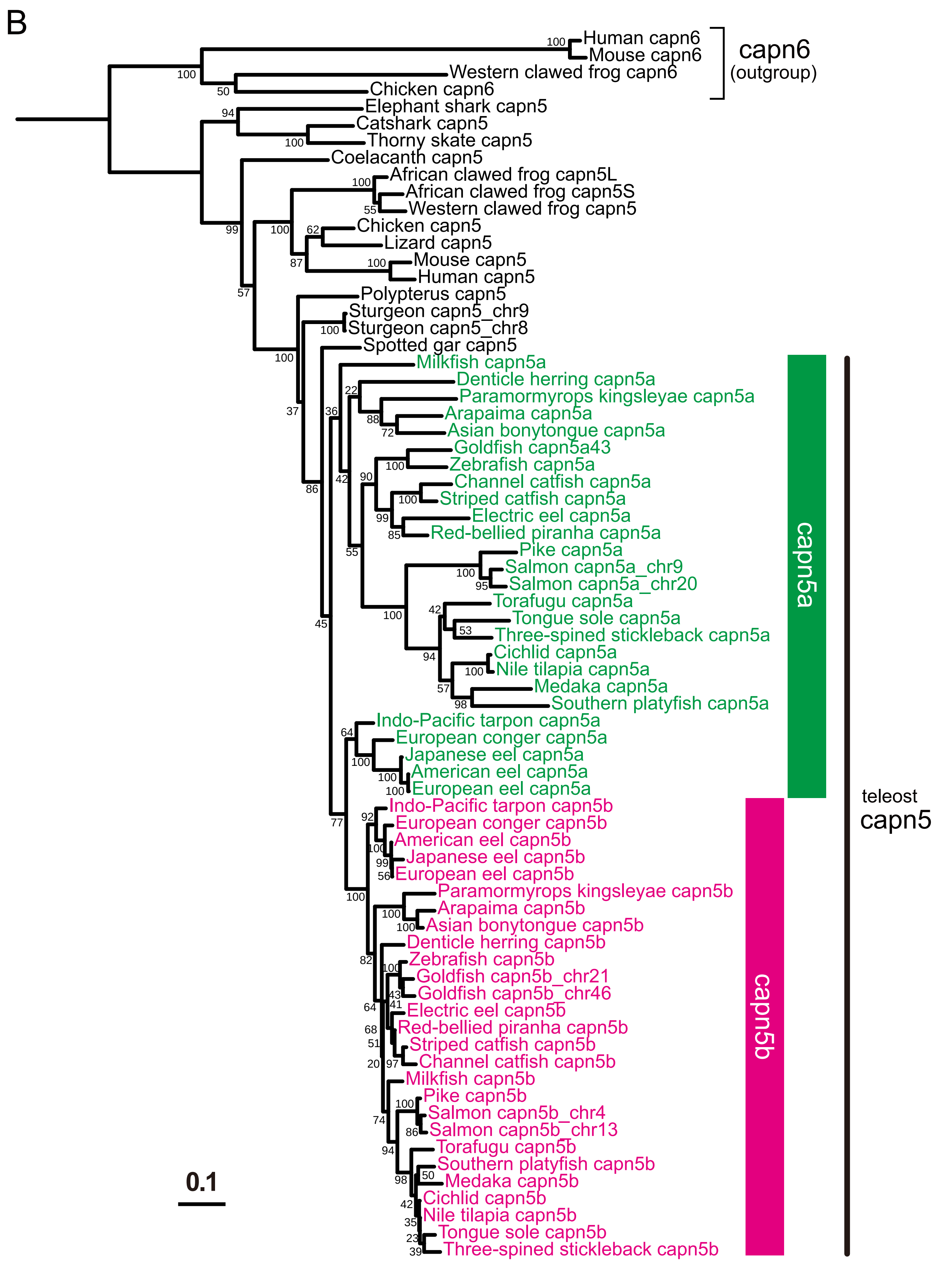

Figure S1B. Detailed maximum-likelihood phylogenetic tree of *capn5* genes shown in Figure 1.

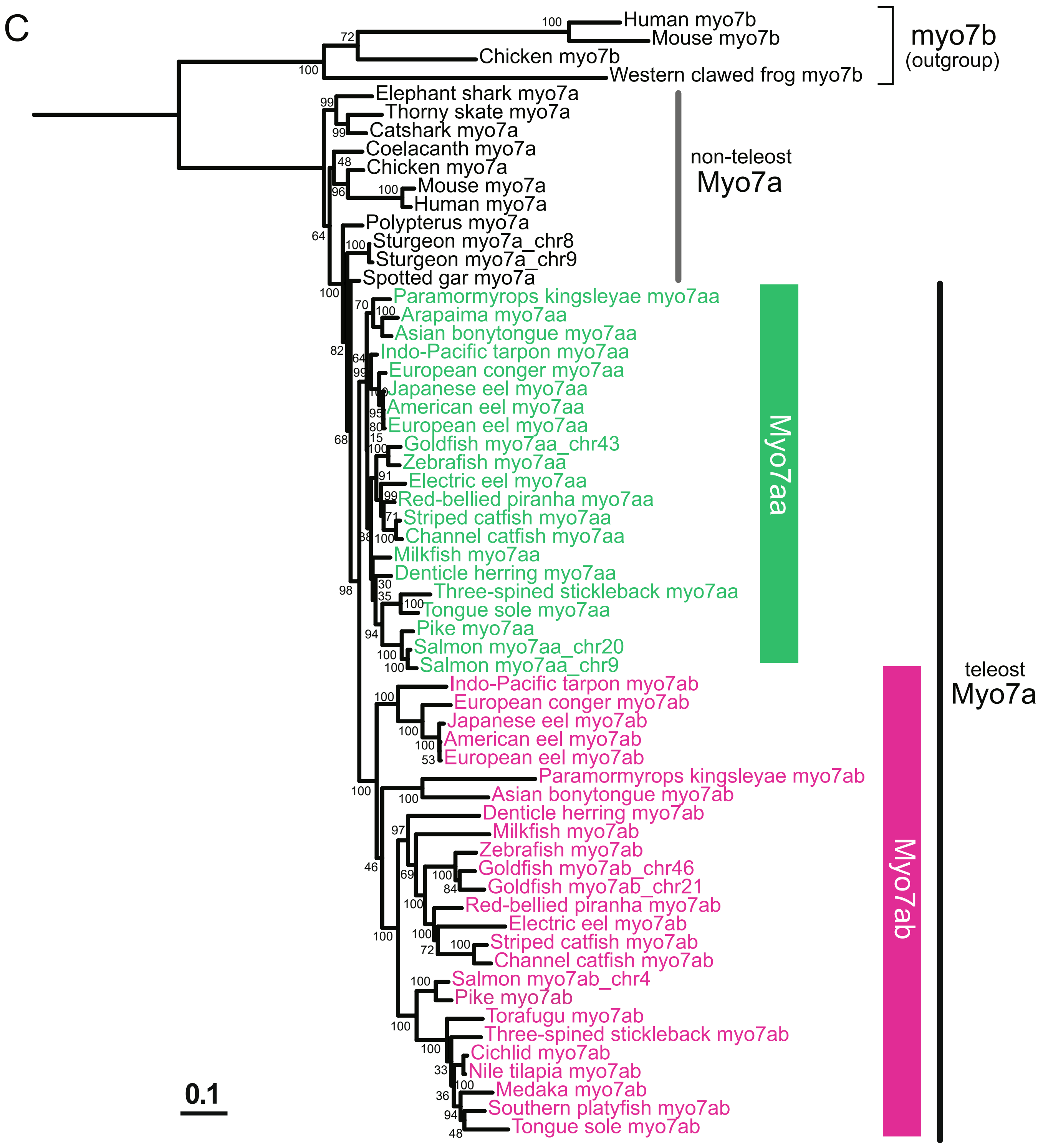

Figure S1C. Detailed maximum-likelihood phylogenetic tree of *myo7* genes shown in Figure 1.

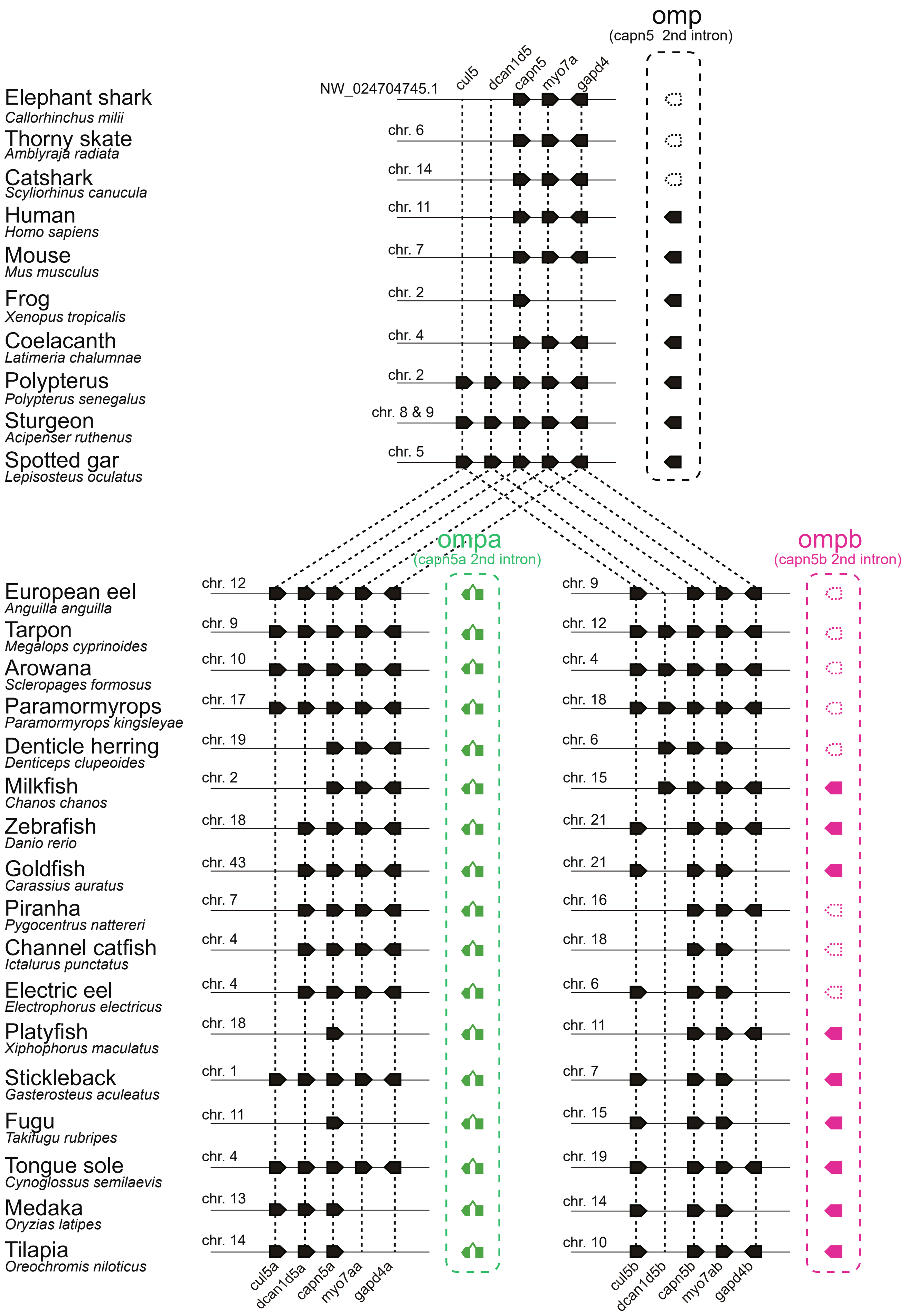

Figure S2. Genomic synteny of *omp* genes

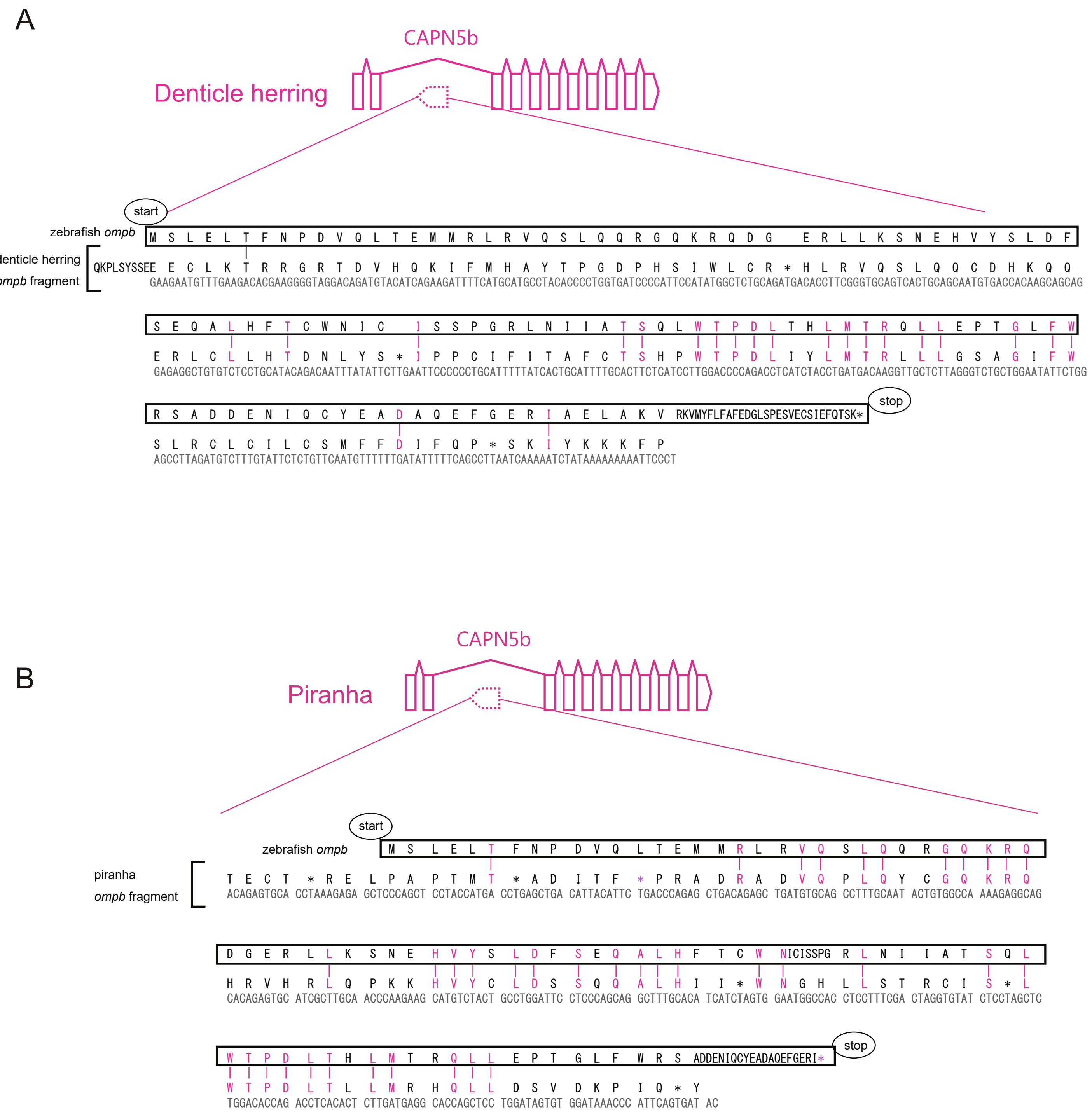

Figure S3. Fragments of pseudogenized *ompb* gene.

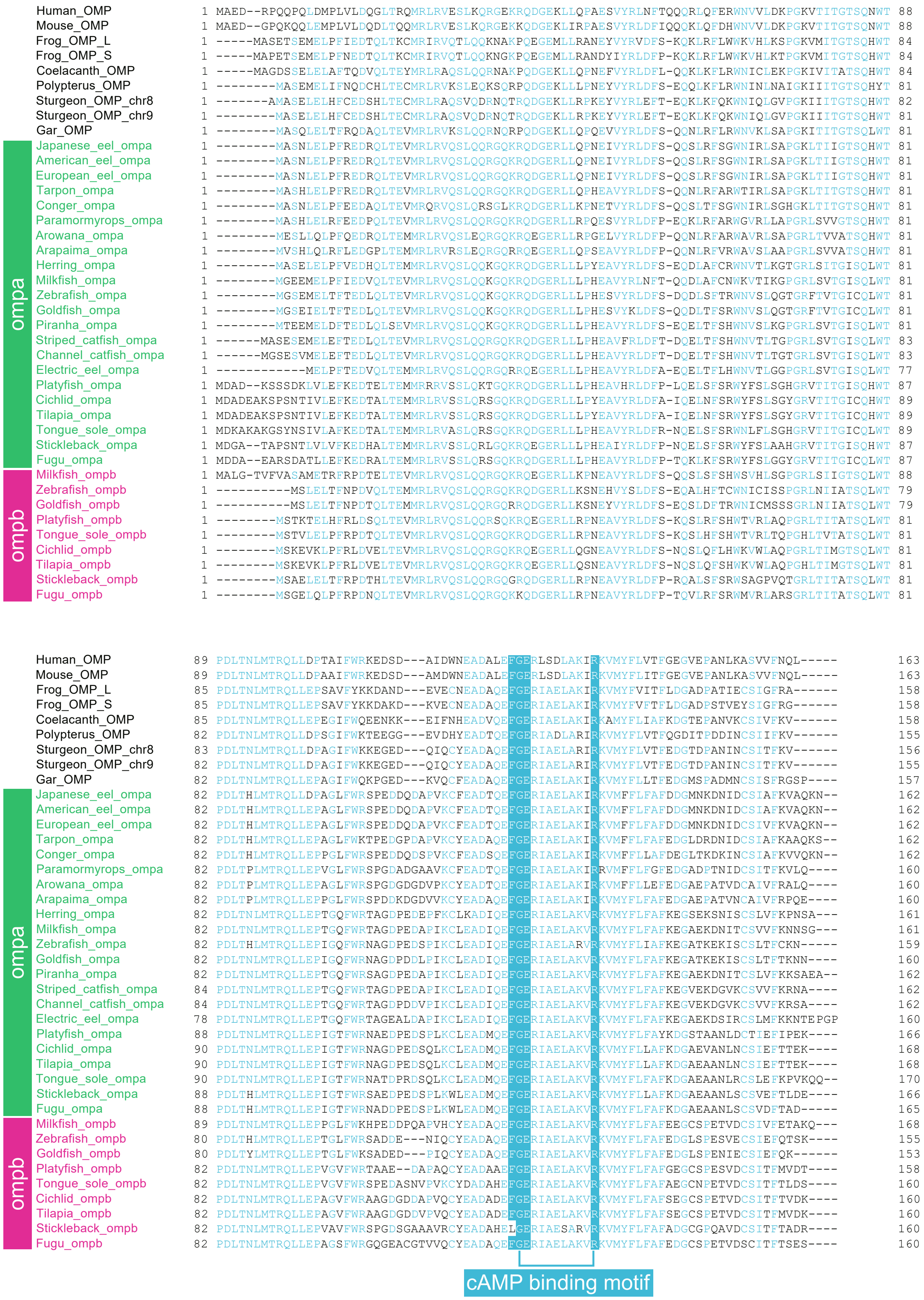

Figure S4. Multiple alignment of *omp* genes and the conserved cAMP-binding motif.

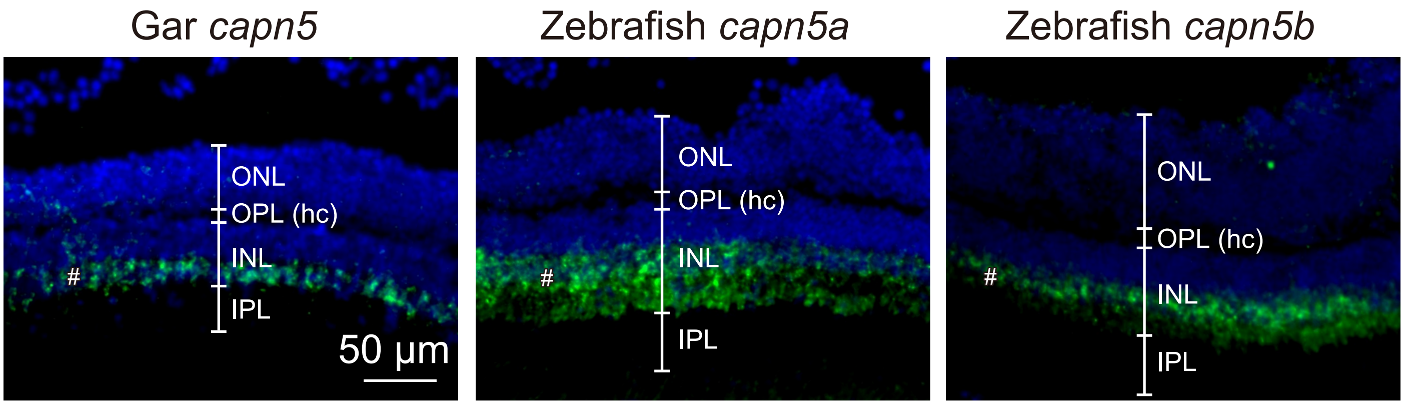

Figure S5. Expression pattern of the *capn5* gene in the retina

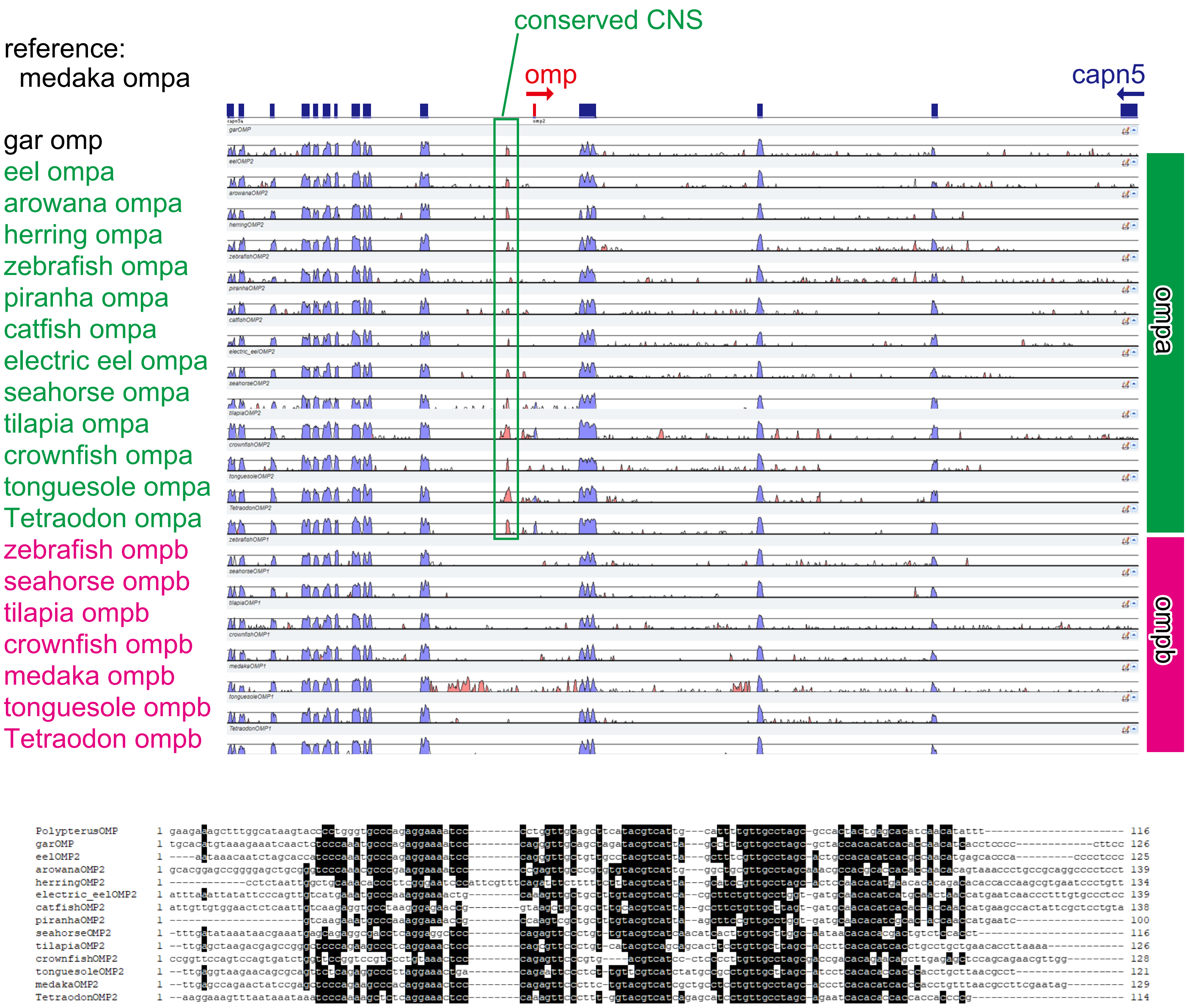

Figure S6. Conservation of upstream regions of *omp* genes revealed by VISTA analysis

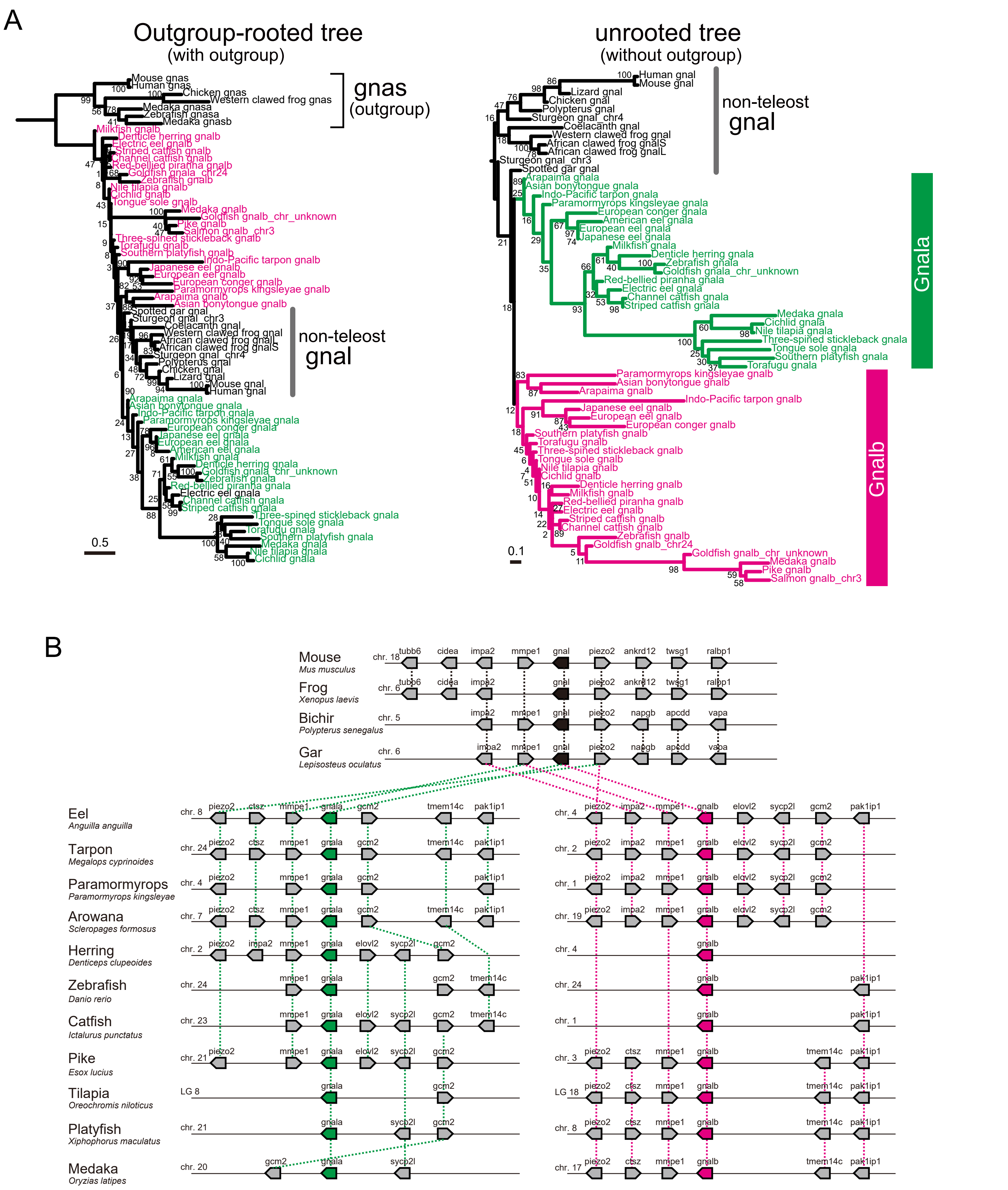

Figure S7. (A) ML phylogenetic trees and (B) genomic synteny of olfactory signaling cascade genes, *gnal*.

C

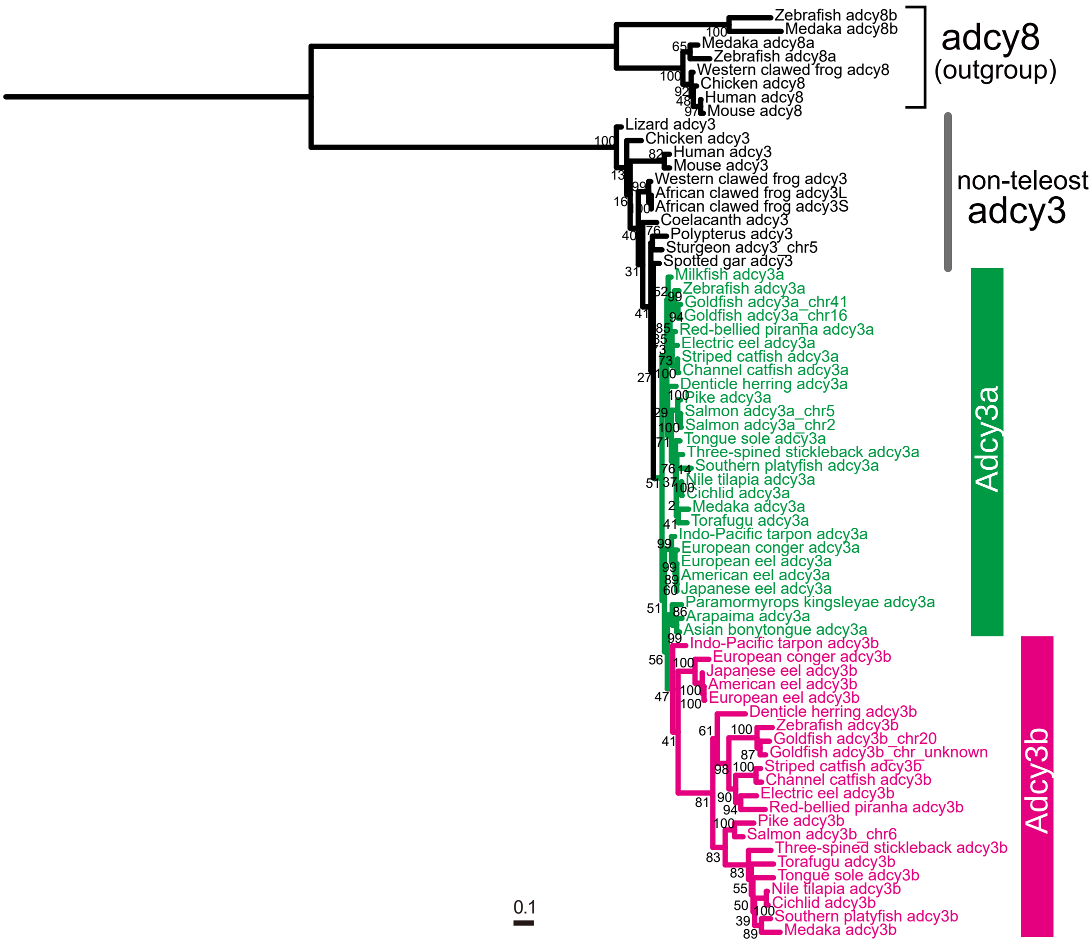

D

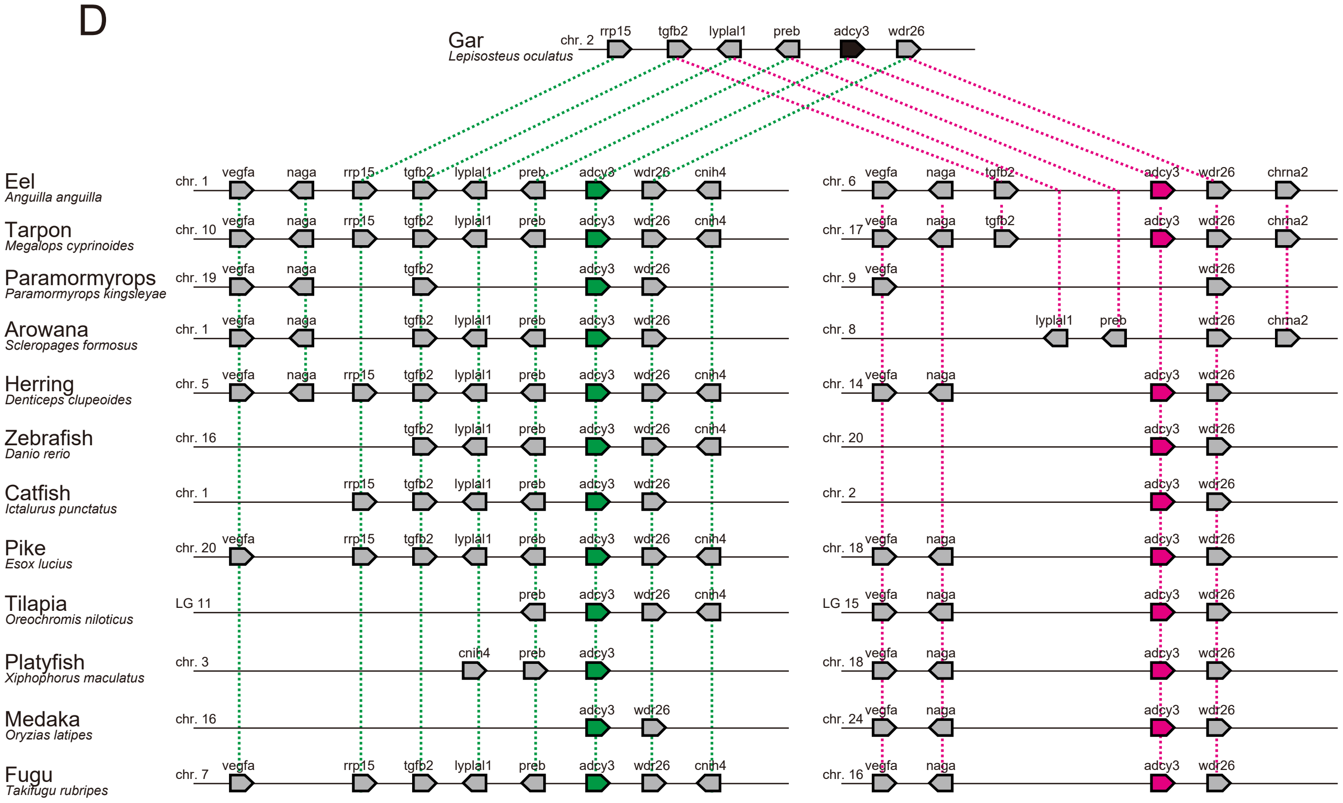

Figure S7. (C) ML phylogenetic trees and (D) genomic synteny of olfactory signaling cascade genes, *adcy3*.

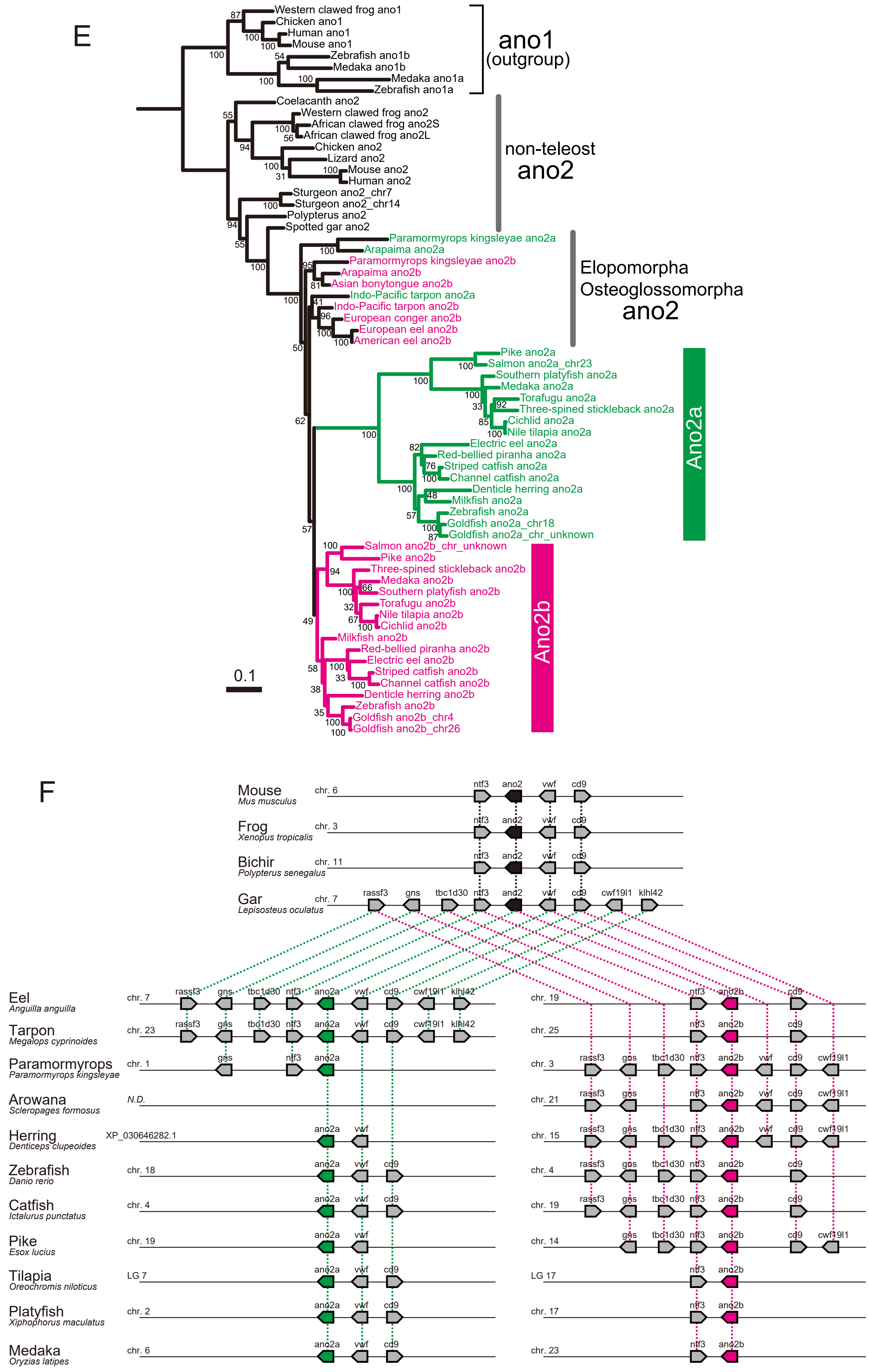

Figure S7. (E) ML phylogenetic trees and (F) genomic synteny of olfactory signaling cascade genes, *ano2.*

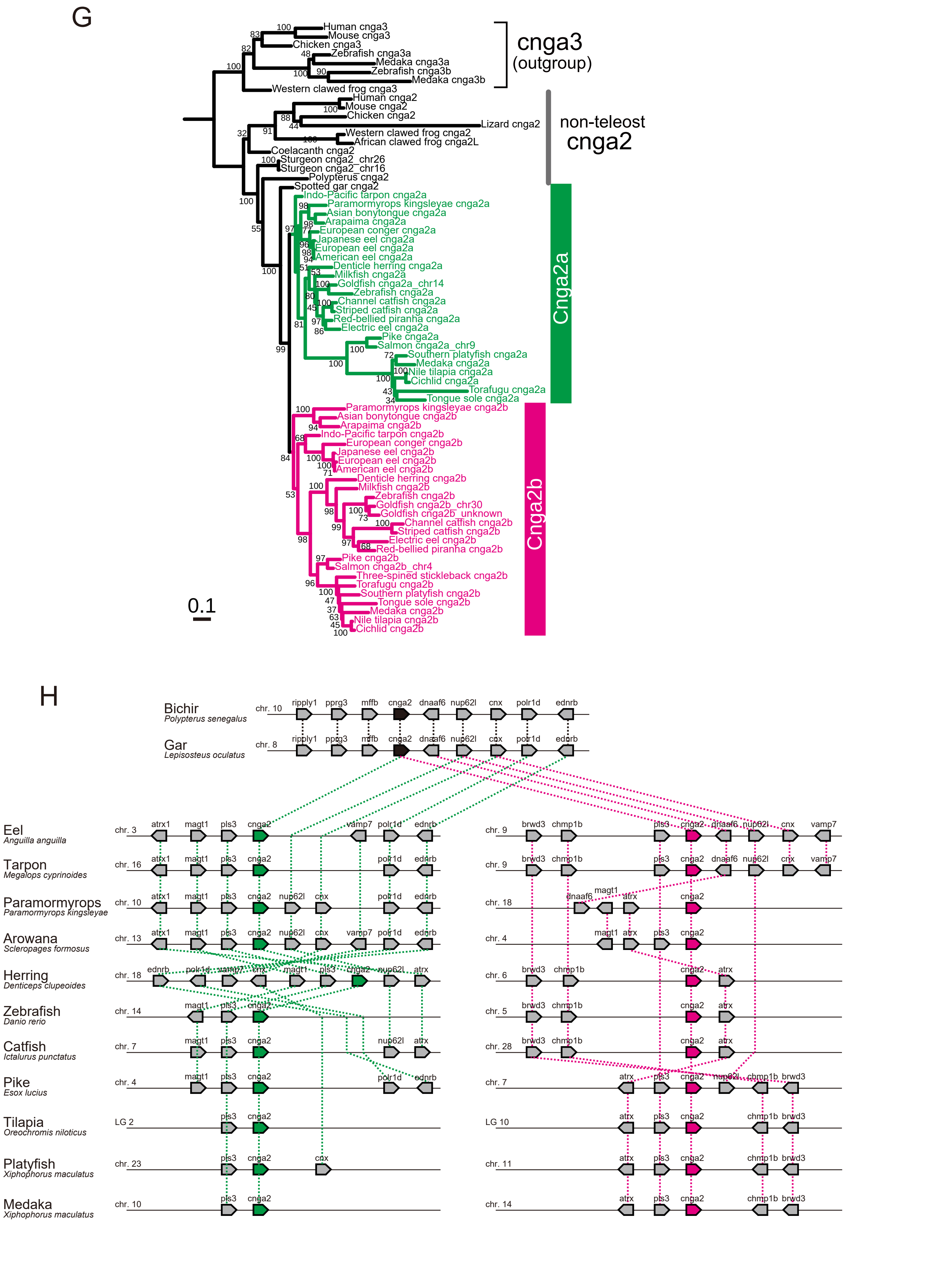

Figure S7. (G) ML phylogenetic trees and (H) genomic synteny of olfactory signaling cascade genes, *cnga2.*

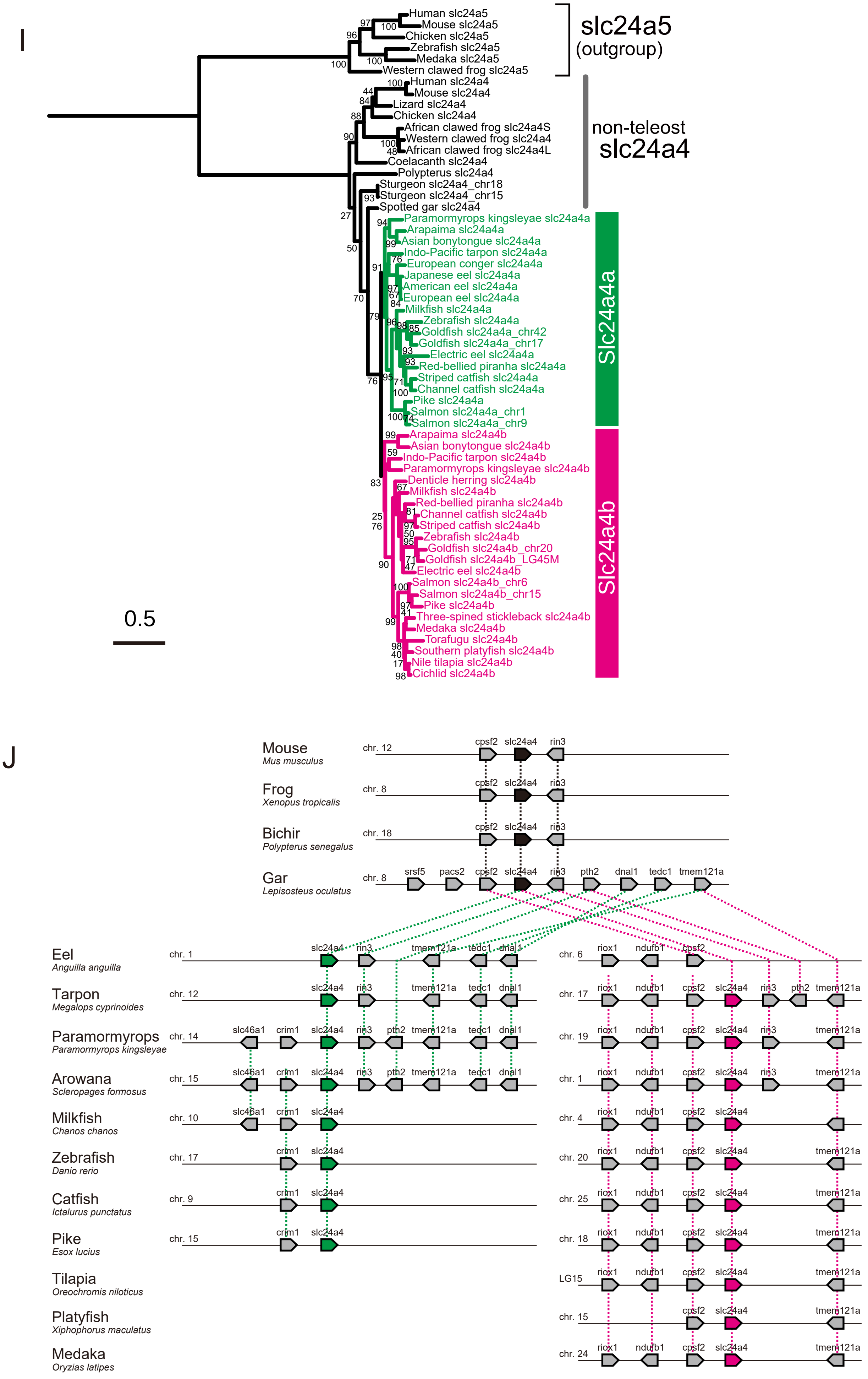

Figure S7. (H) ML phylogenetic trees and (I) genomic synteny of olfactory signaling cascade genes, *slc24a4.*

>DreOmpa_upstream
GAGGTCGACGGTATCGATAAGCTTTCTAGAAAGTGTTGAAGAAATGGTTAACA
GACCAAACGTTTTGCAGTGGCCTAGCCAGAGTCCTGACTTTAACCCAACTGAG
AATCTGTAGAGGAAGCTAATGCAATTCATCTTTATTTTTATAGTGCTTTTATAA
GATTATGTAGATTATGGCAAAGCAGCTTCACATAGAAGATTATAGTGATAAAGA
TCAGGCTGATGGTAAACTCTACAACCTAAAAGAGTAGAAGCTCATCGATAAAG
ATAAATGGGCAAAAATACAAGTGGAGACATGCAGAAAGCAGCTCAGCAATTAT
AGGATGTGTTTGATTGCTATAAATAGCCAAAACAGGATATTGATCATTGAAAA
GGGTATGAATAATTTTGGACAGGACACTTTTTGTTCAAATGTAAATAAAATCT
GTGAAATTAGTTGGTTTTTTCCACAACGATGACTCTTGTCAACGTCTTATTAT
CTTTTGGGAGAAGCCAGTGTCATATTTGGTCAAAAACAACAACTTGCATCAGA
ATTATTCAACACCAGAATTTGCTTAACTGTATATATTCCAGCTAAACAAAAAGT
AGTAAAACCTTCTCCAGAAGACAAATACAACAGGACATTTTGTTATATATTAGG
GGTGTCAAAATTAATTGTCGGTGCACCGCGATGCAGACGCGGACAATTCGGT
ATTGATTCAGTAATAACCATAACCGGTTATTATGTAGTGACGTCATTTATCTCC
TATGCGCTCTGTCGCGAGGGAGGTGAGCGCCATTATTTACAACACTCAGCCA
ACTTGGGGCCGGCCCGTGGCATAGGCACCATAGGCAAATGCTTAGGGCGCTG
TATATCCAGTGGGGCGCCAGAAATGAGCGCGCTTCAGTTGGTTTTGTTTTTG
ATTTTCCTGACACACATTCAGTTATAGACGGCAATAACTCAAAAAAACGCTTAC
CCTAAAAAGATCTAAAGTGTTTCCTACAAAAAGACAAAGCTGGTTATGTTTCA
CAGCTGTCTTCGCTCGACATTACGAGCACTGCTCCGTTTAAGACCTCTCAGTC
TGCGTTTACTTTAGCCGGCAGAGCTCGCGCTGAGAGGAGCTCTCTGTAAGGG
ACCGTCGGAAAAGTGCTTATTTTTTTCGCTGCTCCGCTAAGCGCAAGCTCTC
CCTGTTGCAAGGGCTTAAGTGTGTTTGTTTTTGTTTGTTTGATGCTGTCTAT
GTTTGTGAATGTGTTTGAATGAGAGACAGTGTGTTGCTGTGTGTCTGTGTGT
TTATACAGACAGCATGTTATAGCCTCCCCCCTAAAATATAACACTATATATGGA
AAGCATCGTCAATGCACCGTGATGCACCAAGATATGGAATTGAACCGAATCGA
TGGCATGATAATCGTAACCGAACCGAACTGTGAGACAAGTATAGGTTCACACC
TCGGAAATTGTGAATAAAATTACAATTTTACCTCAAATGCACAAAACAAAGCAA
AACAGAAAGTCTGGAAGTTTTCCAACAGCAGAAAGCATCTGTTGCCTAGAGCT
GCAACACTGAATCTGTTTGCTTTGCTCCTCCTCCTTTTTCCAAAACTAAAGTC
GAGTCTCAGAGAATCAATAAAGTCCCCATCTGAGCGAGTGTGTGTGCCCGAG
GGGACGCAGTGGCGCTGCAGCGGATGGAAAAGTGAAGGCTGAGCGAGGAAA
AGCTTCATTCACACACAGAGAGAATCAGCAAAACAGCATCCCGCGGGATCCA
CCGGTAAGTCGCCACCA

Figure S8. Modified upstream sequence of zebrafish *ompa* inserted into the Venus vector.

Figure S1. Detailed maximum-likelihood phylogenetic trees shown in Figure 1.

Detailed views of the maximum-likelihood phylogenetic trees for (A) *omp*, (B) *capn5*, and (C) *myo7a* shown in Figure 1. Bootstrap values are indicated on each node (values below 50% are shown as multifurcations).

Figure S2. Genomic synteny of *omp* genes.

Schematic representation of the genomic synteny surrounding the *omp* (*capn5*) genes in vertebrates. The orientation of pentagons indicates the transcriptional direction of each gene, and orthologous genes across species are connected with dashed lines. The presence or absence of *omp* within the second intron of *capn5* is highlighted in the dotted box (loss of *omp* is shown as an open pentagon with dashed outlines).

Figure S3. Fragments of pseudogenized *ompb* gene.

(A) Herring and (B) piranha show traces of pseudogenized *ompb* fragments, revealed by translating the nucleotide sequences located within the second intron of *capn5b* and comparing them with *ompb* from the closely related zebrafish. The amino acid sequence of zebrafish *ompb* is shown in the upper panel (boxed), whereas the nucleotide and translated amino acid sequences of herring and piranha are shown below. Identical amino acids are highlighted in magenta and connected by lines.

Figure S4. Multiple alignment of *omp* genes and the conserved cAMP-binding motif.

Multiple alignment of amino acid sequences of all *omp* genes analyzed in this study. Gaps are indicated by dashes, and identical residues are shown in blue. The putative cAMP-binding motif suggested by previous studies [4033] is highlighted with a blue background.

Figure S5. Expression pattern of the *capn5* gene in the retina

Identification of retinal layers was based on a previous study (Yazulla and Studholme, *J. Neurocytol.* 2002). ONL: outer nuclear layer; OPL: outer plexiform layer; INL: inner nuclear layer; IPL: inner plexiform layer. All *capn5* genes were expressed not in the horizontal cells (hc), where the nested gene *omp* is expressed, but rather in the INL. Green fluorescence observed in photoreceptor layers, indicated by (#), is interpreted as background signal rather than specific gene expression. Scale bars represent 50 μm in all panels.

Figure S6. Conservation of upstream regions of *omp* genes revealed by VISTA analysis.

A conserved non-coding sequence was detected upstream of *ompa* across multiple species (highlighted), whereas no comparable conserved region was identified upstream of *ompb*. The region shown includes the promoter fragments used in reporter assays. A multiple sequence alignment of the

conserved region is shown below.

Figure S7. ML phylogenetic trees and genomic synteny of olfactory signaling cascade genes.

(A and B) *gnal*, (C and D) *adcy3*, (E and F) *cnga2*, (G and H) *ano2*, and (I and J) *slc24a4*. Bootstrap values are indicated for all nodes in each phylogenetic tree. Genomic synteny diagrams were illustrated in the same manner as described in Figure S2. For *gnal* (A), both outgroup-rooted and unrooted trees are shown. In the outgroup-rooted tree using *gnas* as an outgroup, non-teleost *gnal* sequences are placed within the teleost clade, likely reflecting the large evolutionary distance between *gnal* and *gnas*. Therefore, an unrooted tree is also presented to illustrate the separation of gnal paralogs. Notably, in both representations, *gnal* duplicates are clearly resolved into two distinct clades.

Figure S8. Modified upstream sequence of zebrafish *ompa* inserted into the Venus vector.

The modified upstream sequence of the zebrafish *ompa* gene inserted into the Venus vector is shown in FASTA format.
